# Structural Analysis of Free *N*-Glycans in α-Glucosidase Mutants of *Saccharomyces cerevisiae*: Lack of the Evidence for the Occurrence of Catabolic α-Glucosidase Acting on the *N*-Glycans

**DOI:** 10.1371/journal.pone.0151891

**Published:** 2016-03-24

**Authors:** Tanim Jabid Hossain, Yoichiro Harada, Hiroto Hirayama, Haruna Tomotake, Akira Seko, Tadashi Suzuki

**Affiliations:** 1 Glycometabolome Team, Systems Glycobiology Research Group, RIKEN-Max Planck Joint Research Center for Systems Chemical Biology, RIKEN Global Research Cluster, Wako, Saitama, Japan; 2 Graduate School of Science and Engineering, Saitama University, Sakura, Saitama, Japan; 3 Japan Science and Technology Agency (JST), ERATO Ito Glycotrilogy Project, Wako, Saitama, Japan; George Washington University, UNITED STATES

## Abstract

*Saccharomyces cerevisiae* produces two different α-glucosidases, Glucosidase 1 (Gls1) and Glucosidase 2 (Gls2), which are responsible for the removal of the glucose molecules from *N-g*lycans (Glc_3_Man_9_GlcNAc_2_) of glycoproteins in the endoplasmic reticulum. Whether any additional α-glucosidases playing a role in catabolizing the glucosylated *N*-glycans are produced by this yeast, however, remains unknown. We report herein on a search for additional α-glucosidases in *S*. *cerevisiae*. To this end, the precise structures of cytosolic free *N*-glycans (FNGs), mainly derived from the peptide:*N*-glycanase (Png1) mediated deglycosylation of *N-*glycoproteins were analyzed in the endoplasmic reticulum α-glucosidase-deficient mutants. 12 new glucosylated FNG structures were successfully identified through 2-dimentional HPLC analysis. On the other hand, non-glucosylated FNGs were not detected at all under any culture conditions. It can therefore be safely concluded that no catabolic α-glucosidases acting on *N*-glycans are produced by this yeast.

## Introduction

**Fig 1:**
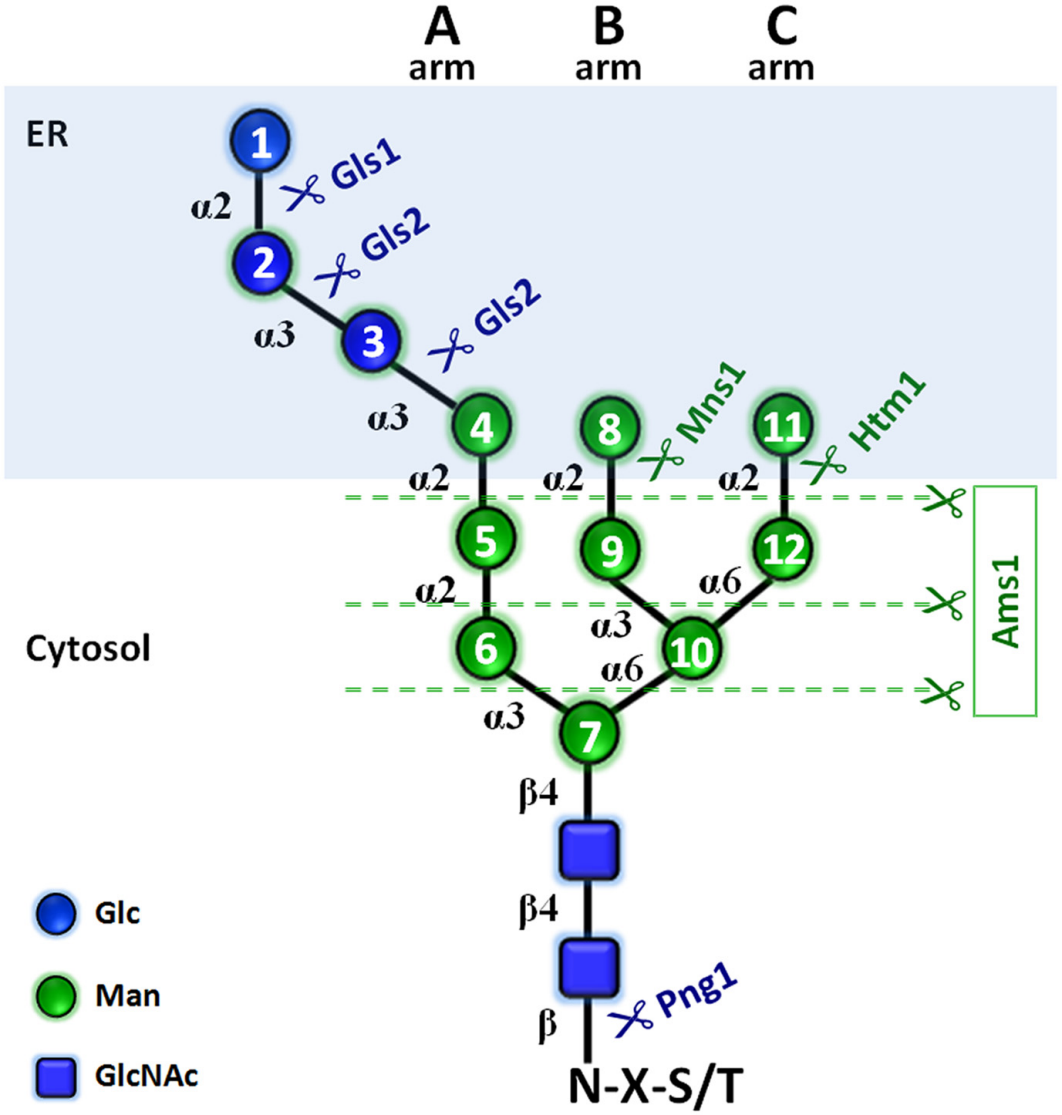
Enzymes that act on *N*-glycans in *S*. *cerevisiae*. The enzymes in the top panel (shaded light blue) are located in the ER. α and β indicates glycosidic linkages. Mns1, ER (α-1,2) mannosidase 1; Htm1, homologous to mannosidase 1.

Asparagine (*N*)-linked glycosylation is an essential modification of proteins going through the secretory pathway, and the reaction is conserved across all three domains of life [1]. For the most part, the biosynthetic pathways leading to *N*-glycosylation are well understood in both the mammalian and yeast cells [2,3]. *N*-glycosylation is mediated by the oligosaccharyltransferase (OST) enzyme catalyzing the *en bloc* transfer of the glycan, Glc_3_Man_9_GlcNAc_2_ in mammals and yeast, from a lipid carrier to the selected asparagine residue of the acceptor polypeptide [3,4]. Two α-glucosidases, glucosidase 1 (Gls1 in *Saccharomyces cerevisiae*) and glucosidase 2 (Gls2), which are located in the endoplasmic reticulum (ER), then quickly remove the glucose molecules from the *N*-glycans (Fig 1). Glucosidase 1 is an ER membrane protein with a lumenal catalytic domain whereas glucosidase 2 is a lumenal protein-heterodimer of two subunits, Gls2 and Gtb1, with the catalytic domain residing in Gls2 [5,6]. Their reactions are believed to play a major role in maintaining the quality control of the newly synthesized glycoproteins in the ER, as their deletion causes a delay or failure in the degradation of certain misfolded glycoproteins [7,8]. As a part of this ER quality control of glycoproteins, the *N*-glycans in various organisms undergo drastic structure remodelling in the ER [9]. The reactions catalyzed by the above α-glucosidases constitute one of their structural remodelling processes in the ER and are well conserved among organisms in which Glc_3_Man_9_GlcNAc_2_ is utilized as a donor substrate for OST. For this reason, the two ER α-glucosidases are widely regarded as “processing” enzymes, rather than catabolic ones, acting on the *N*-glycans.

In sharp contrast to the case of their biosynthesis, many issues remain to be clarified regarding the catabolic processes associated with *N*-glycoproteins [3,10]. For example, as of this writing, only two enzymes, the cytosolic peptide:*N-g*lycanase (Png1) and the cytosol/vacuole α-mannosidase (Ams1), are known to be involved in the catabolic pathway of the *N*-glycans of glycoproteins in *S*. *cerevisiae*. The glycoproteins that fail to acquire their native structures are retrotranslocated from the ER to the cytosol where Png1 removes the *N-g*lycans from the proteins, thus producing the free *N*-glycans (FNGs) [11]. This Png1-catalyzed deglycosylation accounts for >95% of the FNGs observed in this yeast [12,13]. Ams1 then trims the FNGs to eventually form the trisaccharide Man_1_GlcNAc_2_ (Manβ1,4GlcNAcβ1,4GlcNAc) as a final product of the enzymatic catabolism of the FNGs [14–16].

Removal of the three glucose molecules by the ER α-glucosidases has been shown to be critical for the efficient catabolism of the FNGs [13,16]. This may not be so surprising, since the presence of any of these glucose molecules would prevent Ams1 from acting on the A-arm mannose molecules of the *N*-glycans (Fig 1). Interestingly, however, Chantret *et al*., in a previous study, detected the formation of Man_1_GlcNAc_2_ in glucosidase deletion mutants of *S*. *cerevisiae* [16]. This observation suggests that one or more catabolic α-glucosidases that act on the *N*-glycans may be produced by this yeast. In mammalian cells, it has been well established that the inhibition of the ER α-glucosidases can be bypassed by the action of an endo-α-mannosidase in the Golgi [17–20]. *S*. *cerevisiae*, however, does not possess the gene orthologue of endo-α-mannosidase [21], and, consistent with this finding, enzyme activity for this enzyme was not observed [21], making the occurrence of this enzyme unlikely in this yeast.

In this study, we carried out a precise structural analysis of FNGs at different stages of growth in three glucosidase deletion mutants of *S*. *cerevisiae*, *i*.*e*., *gls1*Δ, *gls2*Δ, and *gls1*Δ *gls2*Δ in order to investigate the presence of any catabolic α-glucosidase in this organism. To our surprise, however, no deglucosylation products of the FNGs were detected in any of these cells under all conditions examined. Our results thus indicate that no novel catabolic α-glucosidase acting on the *N-*glycans may be produced by *S*. *cerevisiae*.

## Material and Methods

### Strains and growth conditions

The yeast strains used in this study are listed in Table 1. The deletion mutants were generated by the PCR-based gene deletion technique [22,23]. The primers used for the deletion cassettes can be provided upon request. The cells were grown and maintained in YPD medium (1% yeast extract, 2% peptone, 2% glucose) and all cultures were performed on a rotary shaker at 180 rpm at 30°C unless otherwise noted. Cell growth was monitored by measuring the optical density (OD) at 600 nm. Yeast cells were streaked from a 15% (v/v) glycerol stock onto YPD plate and, after two days of incubation, 15 ml of YPD medium was inoculated with the cells from the plate and cultured overnight. This overnight pre-culture was then inoculated in 200 ml of fresh YPD medium at a cell concentration of OD_600_ = 0.5. After 4 hours (h) and 7 days of incubation, cells equivalent to 30 OD_600_ units were harvested from the culture as log phase (4 h) and stationary phase (day-7) samples.

**Table 1:** Yeast strains used in this study.

Strain	Genotype	Source
*gls1*Δ	*MAT****a*** *his3*Δ*1 leu2*Δ*0 met15*Δ*0 ura3*Δ*0 gls1*Δ::*kanMX4* BY4741	Open Biosystems
*gls2*Δ	*MAT****a*** *his3*Δ*1 leu2*Δ*0 met15*Δ*0 ura3*Δ*0 gls2*Δ::*kanMX4* BY4741	Open Biosystems
*gls1*Δ *gls2*Δ	*MAT****a*** *his3*Δ*1 leu2*Δ*0 met15*Δ*0 ura3*Δ*0 gls1*Δ::*kanMX4 gls2*Δ::*HIS3MX6* BY4741	This study
*ams1*Δ *htm1Δ mns1*Δ *gls2*Δ (*GLS1*^*+*^ strain)	*MAT****a*** *his3*Δ*1 leu2*Δ*0 met15*Δ*0 ura3*Δ*0 ams1*Δ::*HIS3MX6 htm1*Δ::*kanMX6 mns1*::*natMX4 gls2*Δ::*LEU2* BY4741	This study
*ams1*Δ *htm1Δ mns1*Δ *gls1*Δ (*GLS2*^*+*^ strain)	*MAT****a*** *his3*Δ*1 leu2*Δ*0 met15*Δ*0 ura3*Δ*0 ams1*Δ::*HIS3MX6 htm1*Δ::*kanMX6 mns1*::*natMX4 gls1*Δ::*LEU2* BY4741	This study

### Preparation and pyridylamination of FNGs from *Saccharomyces cerevisiae*

The preparation and pyridylamination (labeling with 2-aminopyridine) of the FNGs were carried out as described previously [15].

### Pyridylaminated (PA) glycan standards

PA-G3M9A, PA-G2M9A, PA-G1M9A, PA-G3M8A, PA-G2M8A, PA-G3M8C, PA-G2M8C, PA-G3M7B, PA-G2M7B, PA-G3M9A’, PA-G2M9A’ were prepared as described previously [24–26]. To obtain the demannosylated standard for the glucosylated PA-glycans, the following glycosidase digestions were carried out; digestion of the PA-glycans (2–10 pmol) with Jack bean α-mannosidase (40 mU, Seikagaku Corp.) was carried out in 20 μl of 10 mM sodium citrate buffer, pH 4.0 at 37°C for 16 h. For example, PA-G3M5B was prepared from the treatment of PA-G3M9A by Jack bean α-mannosidase (residue 10 in Fig 1 is resistant to this digestion). Equivalent G2 form of the triglucosylated PA-glycans were prepared by treatment with Gls1-only microsomes (see below).

PA-ManNAc and PA-GlcNAc were purchased from TaKaRa (Kyoto, Japan). Man_1_GlcNAc_1_ManNAc_1_-PA was prepared from wild type yeast BY4741 cells at stationary phase [15]. Briefly, the peak corresponding to Man_1_GlcNAc_2_-PA that also contains Man_1_GlcNAc_1_ManNAc_1_-PA, due to GlcNAc-to-ManNAc epimerization during the PA-labeling reaction [12], was isolated by size fractionation HPLC [15]. The two epimers were then separated and collected by reversed-phase HPLC as described previously [15]. Presence of ManNAc at the reducing end of Man_1_GlcNAc_1_ManNAc_1_-PA was then confirmed by reducing end analysis as previously reported [12] (S1 Fig).

### Microsome preparation

Microsomes were prepared from *ams1*Δ *htm1*Δ *mns1*Δ *gls2*Δ (*GLS1*^*+*^) and *ams1*Δ *htm1*Δ *mns1*Δ *gls1*Δ(*GLS2*^*+*^) yeast cells. The cells were inoculated from a YPD plate to 2 ml YPD medium. After overnight incubation, 1 ml from the culture was transferred to 100 ml of YPD medium and the resulting suspension was incubated at 200 rpm at 30°C. When the OD_600_ of the culture reached 7–10, cells were harvested and washed twice with PBS buffer. Lysis buffer (20 mM HEPES-KOH pH 7.4, 2 mM EDTA, 200 mM sorbitol, 50 mM potassium acetate, 1 mM phenylmethylsulfonyl fluoride) (2 ml) was then added and cells were disrupted with 0.5 mm glass beads using a Multi-beads Shocker (Yasui Kikai, Osaka, Japan). The homogenate was filtrated and then centrifuged at 2,150 × *g* for 5 min. The supernatant was collected and centrifuged again at 6,000 × *g* for 5 min. The supernatant was subjected to ultracentrifugation at 100,000 × *g* for 30 min after which the pellet containing the microsomes was resuspended in 2 ml of the resuspension buffer (20 mM Tris-HCl pH 7.4, 250 mM sucrose, 1 mM phenylmethylsulfonyl fluoride) and ultracentrifuged again at 100,000 × *g* for 30 min. The pellet containing the microsomes was finally resuspended in 200 μl of resuspension buffer, flash frozen in liquid nitrogen and stored at -80°C until use.

### Digestion of PA-glycans with microsomes

The PA-glycans and their isomers were treated with the microsome for deglucosylation. 4 μl of each of Gls1-only and/or Gls2-only microsomes was used in a 20 μl reaction cocktail containing 20 mM HEPES-KOH (pH 7.4), 1% (w/v) Triton X-100, and water and incubated overnight at 37°C.

### Digestion of PA-glycans with endo-α-mannosidase

Presence of the innermost glucose molecule (residue 3 in Fig 1) in the microsome treated FNG isomers was examined by digestion with endo-α-mannosidase. A cDNA encoding the C-terminal catalytic domain of *Branchiostoma floridae* Golgi endo-α-mannosidase (356 amino acids from the C-terminus; accession No. XM_002590288) was cloned into pCold I expression vector (Takara Bio Inc., Otsu, Japan) between *Nde*I and *Xba*I restriction sites, which was designed to produce *N-*terminally (His)_6_-tagged proteins. The plasmid was transfected into BL21 cells possessing pGro7 chaperone plasmid (Takara Bio Inc.), and was grown at 37°C in LB broth containing ampicillin (100 μg/ml) and chloramphenicol (30 μg/ml) and 0.5 mg/ml l-arabinose, until the OD_600_ reached 0.45. The culture was then incubated at 15°C for 30 min, followed by the addition of isopropyl-1-thio-β-d-galactoside to a final concentration of 0.05 mM. The culture was further incubated with agitation (160–180 rpm) at 15°C for 22 h. The cells were harvested and lysed by sonication in 33 ml of BugBuster (Novagen) containing 1 × Complete^™^ protease inhibitor mixture (Roche Applied Science) and 1 mM Pefabloc (Roche Applied Science). The cell lysate was filtrated through 0.45 μm filter (Millipore), and was centrifuged at 11,000 × *g* at 4°C for 2 min, and the supernatant was applied to 2 ml nickel-Sepharose Fast Flow resin (GE Healthcare) preequilibrated with binding buffer (20 mM Tris-HCl buffer (pH 8.0) containing 300 mM NaCl). After the sample was applied, column was washed with 5 volumes (10 ml) of binding buffer containing 25 mM imidazole. Finally the bound enzyme was eluted with 5 volumes (10 ml) of binding buffer containing 500 mM imidazole. Eluted fraction thus obtained was dialyzed against the binding buffer, concentrated using Amicon Ultra-15 3K (Amicon) and was used as an enzyme fraction. The protein concentration of the enzyme fraction was determined by BCA protein assay kit (Pierce) with bovine serum albumin as a standard, according to the manufacturer’s protocol. Enzyme concentration was determined to be 2.6 mg/ml, and fractions were aliquoted and stored at -80°C until use. The enzyme digestion was carried out in 20 μl reaction mixture including 1 μl endo-α-mannosidase fraction and 0.1 M MES-NaOH (pH 6.5), and incubated overnight at 37°C.

To examine if reducing termini of some of the FNGs observed were epimerized to form ManNAc-derivative during PA-labeling, FNGs were first digested with endo-α-mannosidase as described above. The digested FNGs were recovered in 70% ethanol and evaporated to dryness. The dried FNG sample was then used for digestion with the Jack bean α-mannosidase using the same reaction cocktail used for the preparation of PA-labeled glycan standards as described above.

### High performance liquid chromatography (HPLC)

The deglucosylation of PA-G3M9A’ (PA-Glc_3_Man_9_GlcNAc_1_) and PA-G2M9A’ (PA-Glc_2_Man_9_GlcNAc_1_) by the Gls1-only and/or Gls2-only microsomes was checked by size-fractionation HPLC as reported previously [13]. PA-labeled FNGs from the glucosidase mutant cells were separated by size-fractionation HPLC with a Shodex NH2P-50 4E column (4.6 × 250 mm; Shodex), as reported previously [15]. To further separate the isomers of the PA-glycans, each PA-glycan fraction, which had been separated by the size fractionation HPLC, was re-injected in reversed-phase HPLC using a TSK-gel ODS-80TM column (4.6 × 150 mm; TOSOH, Tokyo, Japan) as described previously [15]. FNGs were quantitated from the HPLC profile using standard PA-glucose hexamer (PA-Glc_6_; 2 pmol/μl) in the standard PA-glucose oligomer (degree of polymerization = 3–15; Takara) as a quantitation reference.

### Determination of cell concentration

Cell concentration of yeast under various culture conditions was determined by OD_600_ measurements, as described previously [15].

## Results

### Use of yeast microsome as a source of the glucosidase enzyme(s)

The objective of this study was to determine whether any α-glucosidase other than Gls1/Gls2 is produced by *S*. *cerevisiae* that is capable of acting on the glucosylated *N*-glycans. To this end, we carried out a precise structural analysis of FNGs in ER α-glucosidase-deficient strains, *i*.*e*. *gls1*Δ, *gls2*Δ *and gls1*Δ *gls2*Δ cells. The rationale behind this approach is, if there are catabolic α-glucosidases in yeast, deglucosylated FNGs should be detected in these strains. We faced, however, some technical problems for the determination of glucosylated FNGs; while we identify the structures of FNGs by HPLC-based mapping methods [27], standard PA-labeled glucosylated glycans are not available. Moreover, there is no facile way to remove the α-glucose residues that are attached to FNGs, since neither the ER α-glucosidase 1 and 2 is commercially available. If these α-glucosidases were available, the glucosylated FNGs could be treated with those enzymes and the structures of the deglucosylated FNGs could then be determined using a method previously described [12].

To solve this problem, we developed a method that involved utilizing yeast microsomes as an enzyme source for the ER α-glucosidases. In *S*. *cerevisiae*, there are 5 glycosidases that are known to act on *N-*glycans; Ams1 (cytosol/vacuole α-mannosidase) [12,14]; Mns1 (ER α-mannosidase 1) [28], Htm1 (ER α-mannosidase acting on misfolded glycoproteins) [29], and Gls1/Gls2. We generated strains for which the genes for all α-mannosidases were deleted, and the 4^th^ deletion was generated for either *GLS1* or *GLS2*. We hypothesized that microsomes from the former strain could be used as a source of ER α-glucosidase 2 (“Gls2-only microsomes”), while the latter could be used as a source of the ER α-glucosidase 1 (“Gls1-only microsomes”).

**Fig 2:**
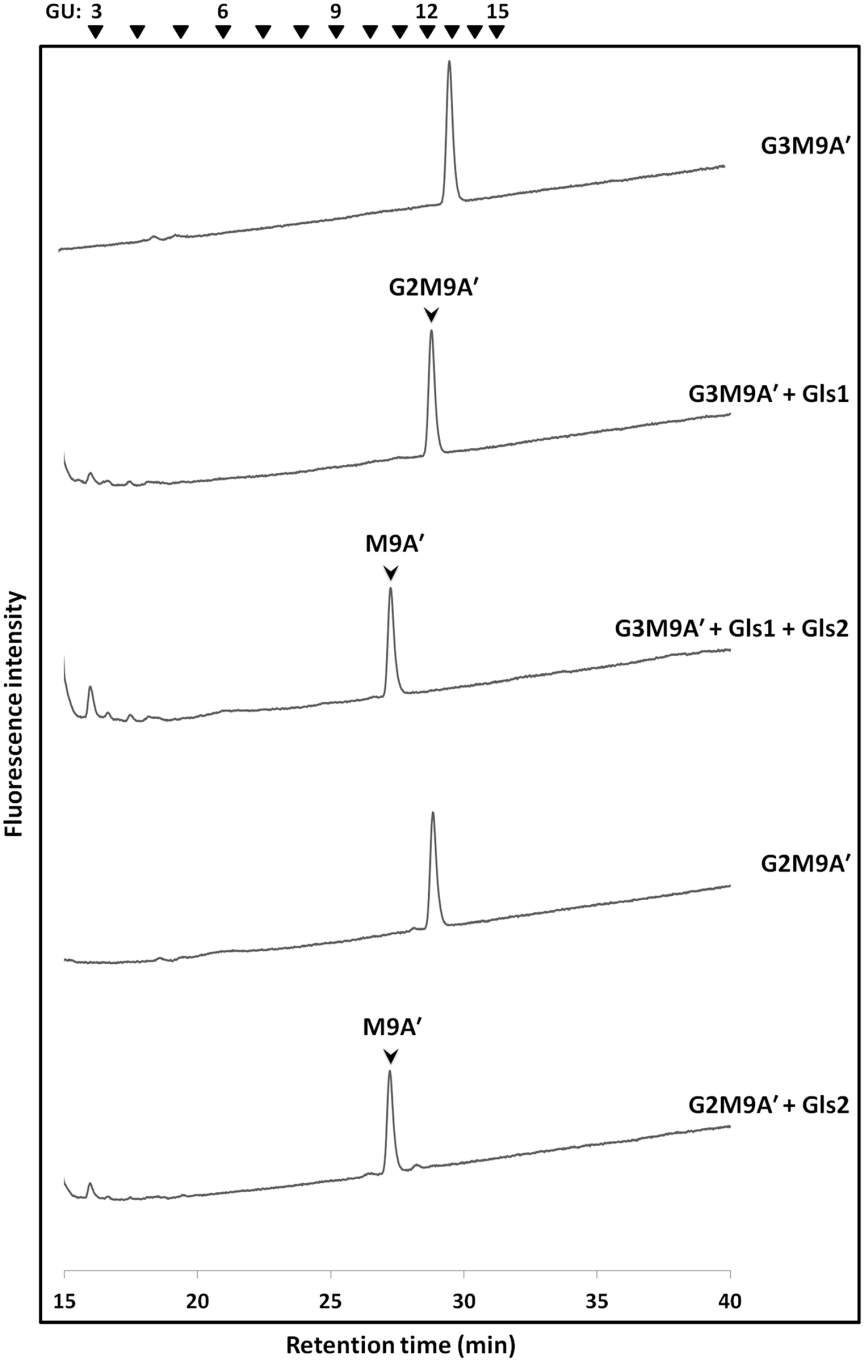
Digestion of PA-labeled glucosylated FNGs with Gls1-only and/or Gls2-only microsomes. Size-fractionation HPLC profiles are shown. The arrowheads indicate the elution position of PA-glucose oligomer for elution standards; (top panel) profile of PA-G3M9A’. (2^nd^ panel) profile of PA-G3M9A’ digested with Gls1-only microsomes. Arrow shows the elution position of PA-G2M9A’. (3^rd^ panel) profile of PA-G3M9A’ digested with Gls1-only and Gls2-only microsomes. Arrow shows the elution position of PA-M9A’. (4^th^ panel) profile of PA-G2M9A’. (5^th^ panel) profile of PA-G2M9A’ digested with Gls2-only microsomes. Arrow shows the elution position of PA-M9A’.

To test our hypothesis, we prepared yeast microsomes from those strains, and incubated them with several standard PA-labeled glucosylated FNGs. When PA-G3M9A’ was treated with the Gls1-only microsomes, its elution position was shifted earlier by one glucose unit (GU) in size fractionation HPLC, indicating that one glucose had been removed from PA-G3M9A’ (Fig 2, compare the first and second panels), whereas treatment with both the Gls1-only and Gls2-only microsomes shifted its elution position earlier by three GUs, indicating that all the three glucoses had been removed (Fig 2, compare first and third panels). Moreover, the Gls2-only microsomes shifted the elution position of PA-G2M9A’ by two GUs, suggesting that two glucoses had been removed from PA-G2M9A’ (Fig 2, compare the fourth and fifth panels). On the other hand, when PA-G3M9A’ was treated with Gls2-only microsomes or PA-G2M9A’ was treated with Gls1-only microsomes, the elution positions of these glycans remained unchanged (S2 Fig), thus confirming that there were no contaminating activities detrimental to the structural analysis of the PA-labeled FNGs. These results clearly indicate that yeast microsomes can be efficiently used as an enzyme source of ER α-glucosidases for the structural characterization of PA-labeled glucosylated FNGs. Our results also suggest that, at least in the microsome fraction, there are no additional α-glucosidases aside from Gls1/2 that can act on glucosylated FNGs.

### Analysis of FNGs in the *gls1*Δ and *gls1*Δ *gls2*Δ cells

**Fig 3:**
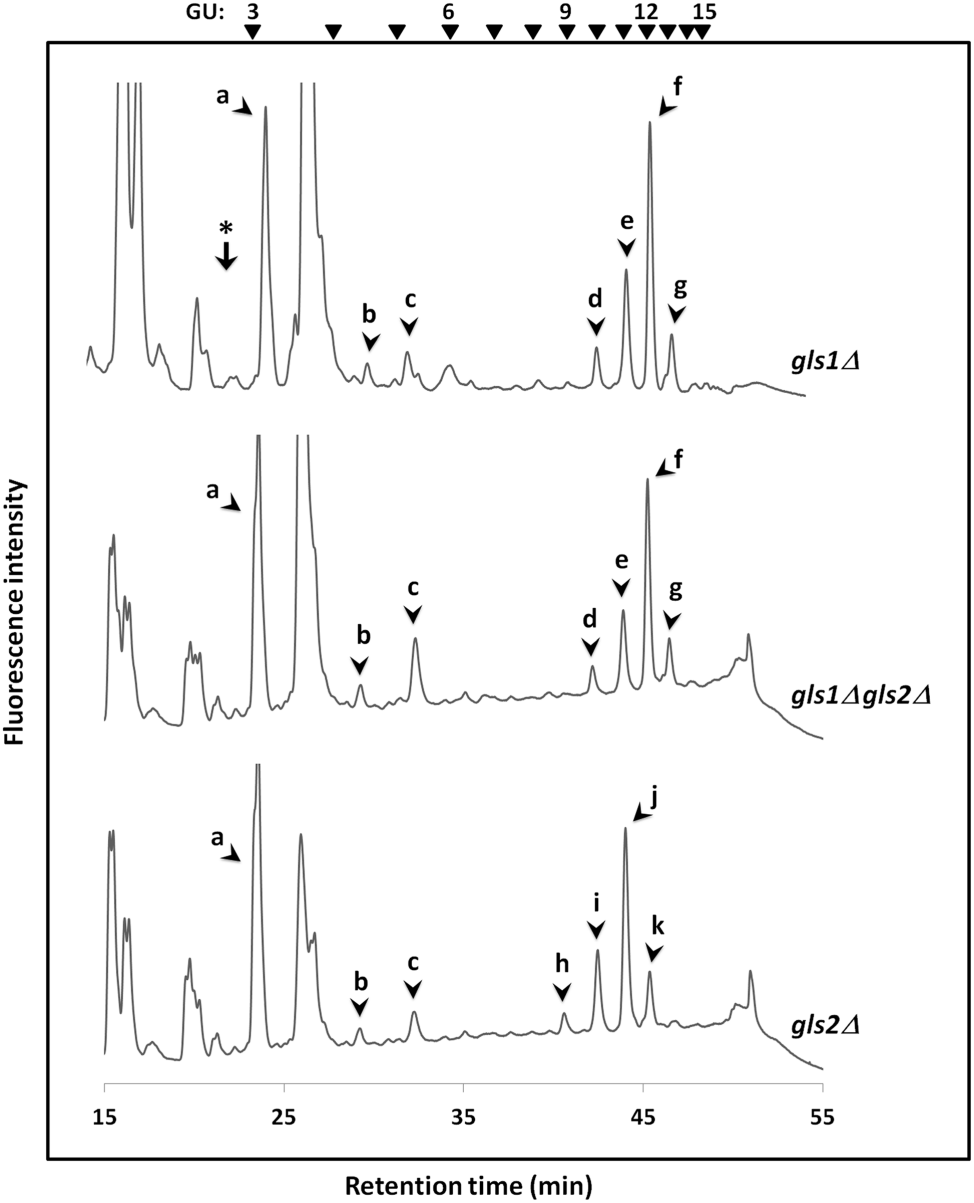
Size-fractionation HPLC profiles of PA-labeled FNGs derived from the glucosidase mutant cells in log phase. Peaks 'a-c' which were observed in all the three glucosidase mutants, are not likely to be FNGs. Peaks 'd-g' produced in *gls1*Δ and *gls1*Δ *gls2*Δ cells, correspond to Glc_3_Man_6-9_GlcNAc_2_-FNGs, whereas 'h-k' from *gls2*Δ cells correspond to Glc_2_Man_6-9_GlcNAc_2_-FNGs. The arrowheads indicate the elution position of PA-glucose oligomer for elution standards. The arrow with the asterisk shows the elution position of Man_1_GlcNAc_2_.

To examine the issue of whether any deglucosylation of the *N*-glycans takes place in the absence of the known α-glucosidases in yeast, first we analyzed FNG structures in the log phase culture of the *gls1*Δ and *gls1*Δ *gls2*Δ cells. FNGs were extracted after 4 h of incubation, labeled with 2-aminopyridine (PA) and analyzed by size-fractionation HPLC. A number of peaks were observed in each of the glucosidase deletion mutants (Fig 3, top and middle panels). The peaks ‘a-c’, which were also observed in our previous analysis with the wild type cells [15], were found resistant to treatment with Jack bean α-mannosidase as well as to the Gls1-only/Gls2-only microsomes (S3 Fig), suggesting that these peaks are not related to *N*-glycans. On the other hand, the peaks ‘d-g’ were all susceptible to the treatment with Gls1-only microsome but resistant to Gls2-only microsome, indicating the presence of the three glucose molecules including the outermost α-1,2 linked glucose. The elution time of the peaks, as compared to the elution time of the GUs, suggests that these peaks correspond to Hex_9-12_GlcNAc_2_-PA. Taken together, these results indicate that peaks ‘d-g’ represent Glc_3_Man_6-9_GlcNAc_2_-FNGs.

**Fig 4:**
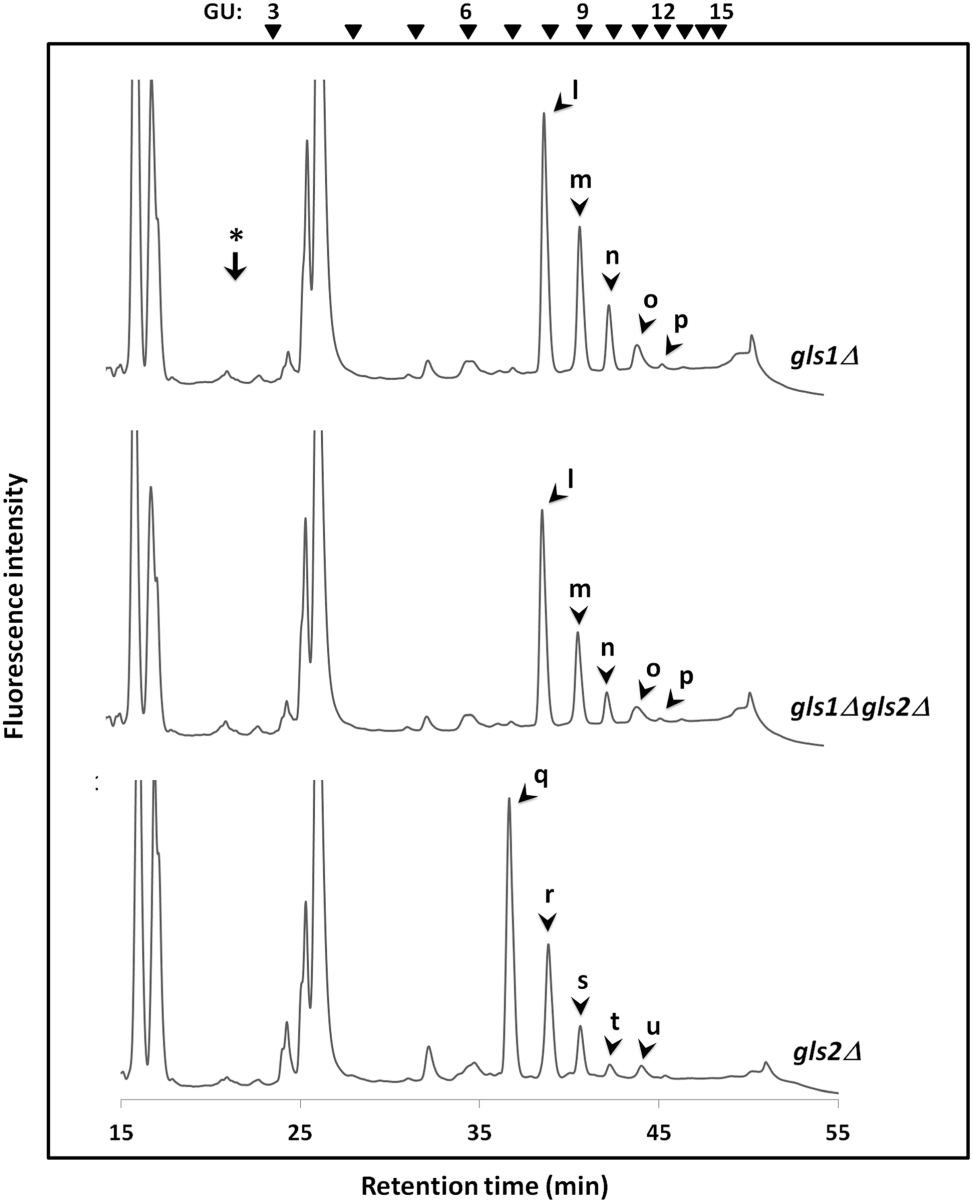
Size-fractionation HPLC profiles of PA-labeled FNGs derived from the glucosidase mutant cells in stationary phase. Peaks 'l-p' produced in *gls1*Δ and *gls1*Δ *gls2*Δ cells, correspond to Glc_3_Man_4-8_GlcNAc_2_-FNGs, whereas peaks 'q-u' produced in *gls2*Δ cells correspond to Glc_2_Man_4-8_GlcNAc_2_-FNGs. The arrowheads indicate the elution position of PA-glucose oligomer for elution standards. The arrow with the asterisk shows the elution position of Man_1_GlcNAc_2_.

It was previously reported that the expression of Png1 and Ams1, the two enzymes involved in the *N*-glycan catabolic pathway, is increased after the log phase, suggesting that the catabolism of *N*-glycans on glycoproteins may be enhanced in post-log phases [30,31]. It is also noteworthy that, in stationary cells, substantial amounts of Man_1_GlcNAc_2_ have been observed [15,16]. In order to explore whether there is any similar stationary phase-specific expression of catabolic α-glucosidase, we then determined the FNG structures in day-7 culture of *gls1*Δ and *gls1*Δ *gls2*Δ cells by HPLC. The elution time with respect to the GUs as well as the results of glucosidase digestion using microsomes, suggested that the peaks ‘l-p’ (Fig 4, top and middle panels) correspond to Glc_3_Man_4-8_GlcNAc_2_-FNGs.

**Fig 5:**
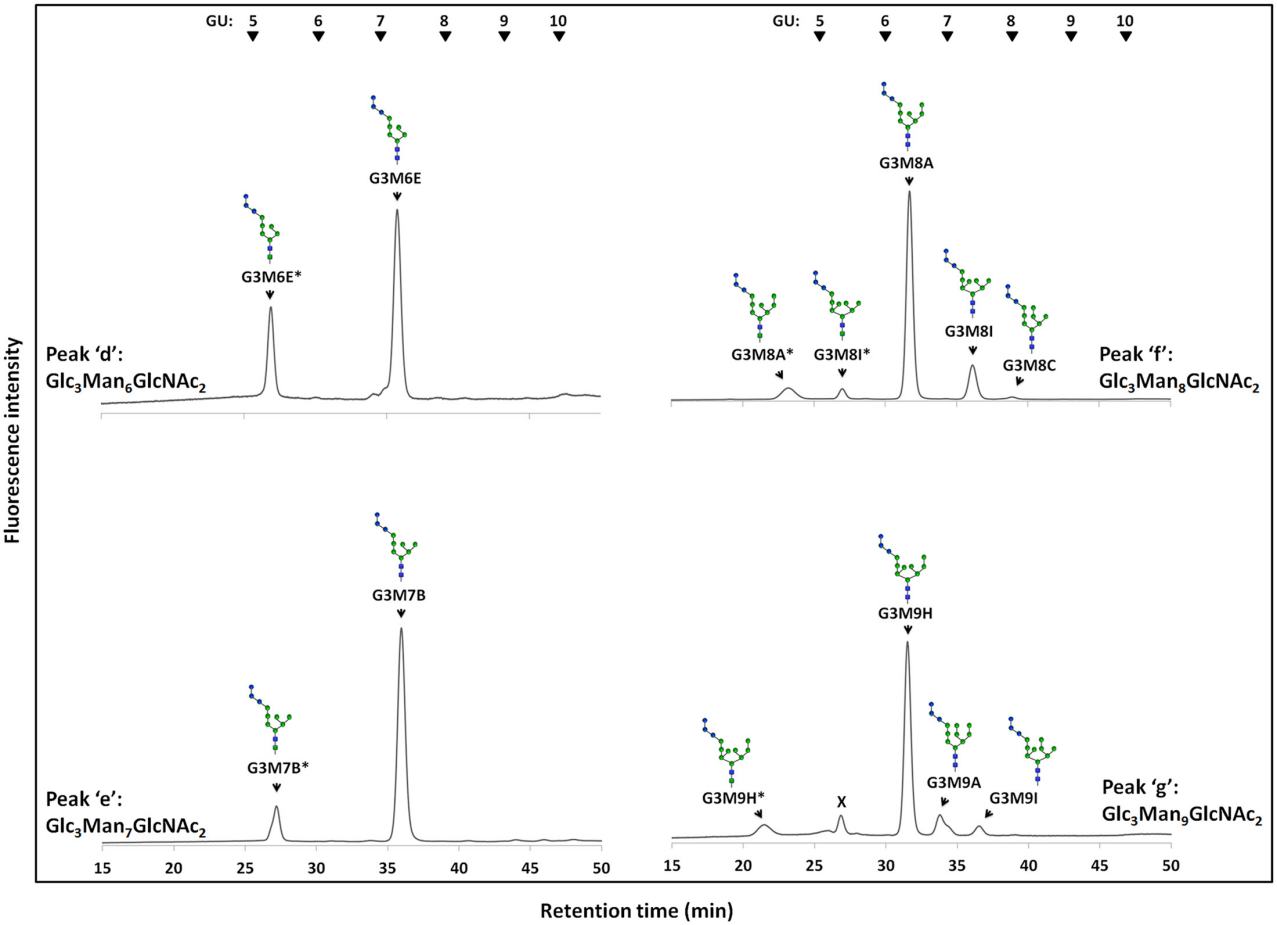
Reversed-phase HPLC profiles of Glc_3_Man_6-9_GlcNAc_2_ in the log phase of *gls1*Δ cells. Peaks 'd-g' of *gls1*Δ cells were isolated by size fractionation HPLC and re-injected in reversed-phase HPLC to separate the isomers of each FNG. Asterisks (*) indicate the structures containing ManNAc residue at their reducing ends. Those ManNAc-containing PA-glycans are produced by GlcNAc-to-ManNAc epimerization of the *N*-acetyl group during PA-labeling reaction. The arrowheads indicate the elution position of PA-glucose oligomer for elution standards. The contaminating peak indicated by × was observed in almost all samples as a very minor component (not seen under normal analytical conditions and only visible when much more sample was injected to detect the very minor FNGs).

**Fig 6:**
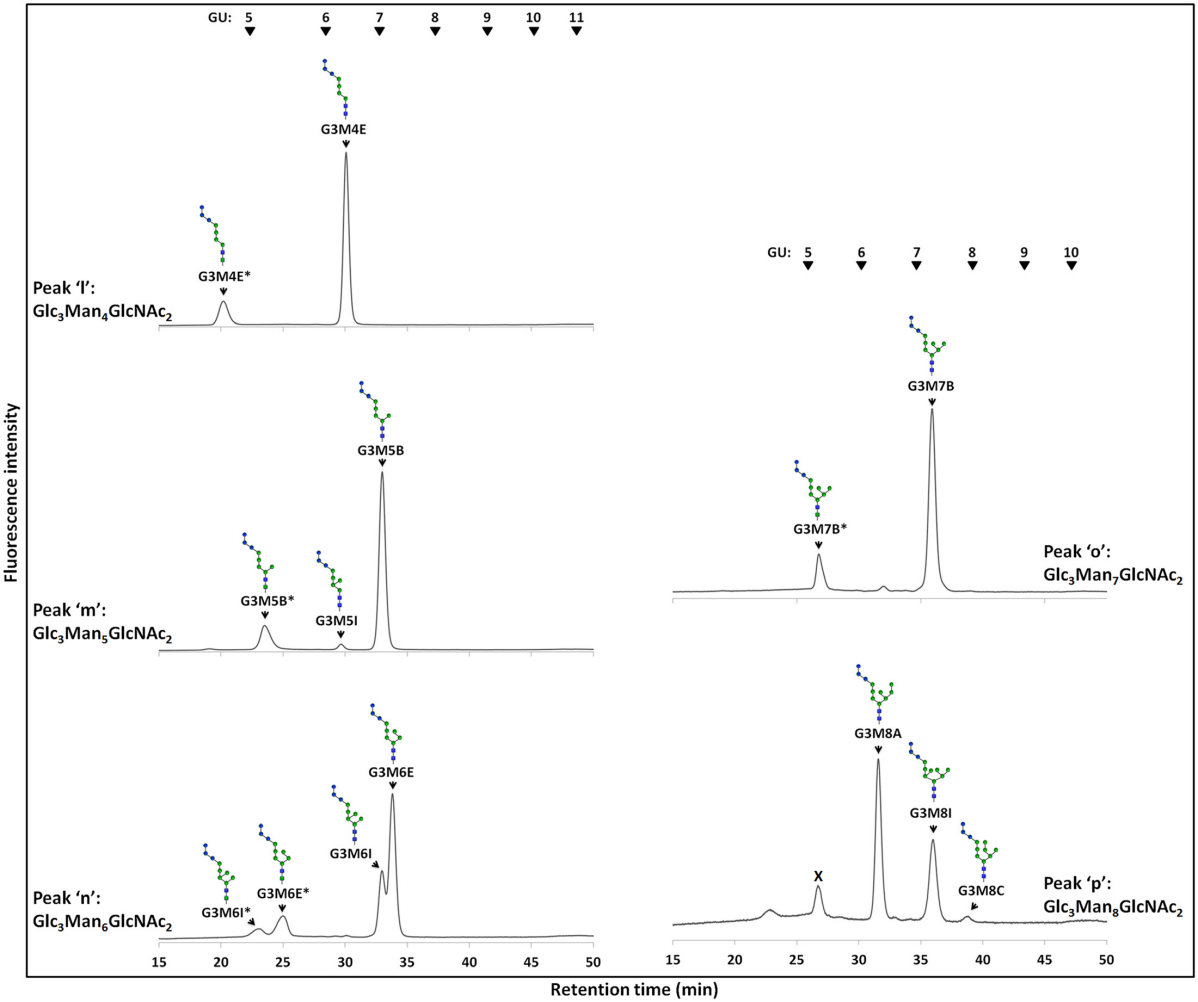
Reversed-phase HPLC profiles of Glc_3_Man_4-8_GlcNAc_2_ in the stationary phase of *gls1*Δ cells. Peaks 'l-p' of *gls1*Δ cells were isolated by size fractionation HPLC and re-injected in reversed-phase HPLC to separate the isomers of each FNG. Asterisks (*) indicate the structures with ManNAc residue at the reducing ends. The arrowheads indicate the elution position of PA-glucose oligomer for elution standards. The contaminating peak indicated by × was observed in almost all samples as a very minor component (not seen under normal analytical conditions and only visible when much more sample was injected to detect the very minor FNGs).

Next, to determine the isomeric structures of these FNGs, the peaks ‘d-g’ and ‘l-p’ (Figs 3 and 4) were isolated by size fractionation HPLC, and the isomers of each FNG were then separated by reversed-phase HPLC (Figs 5 and 6). These methods have been successfully used in previous studies to determine the precise structures of FNGs in wild type or *och1*Δ strains [12,32]. It has been shown that a specific sugar residue shows similar effect on the elution of the *N-*glycans in reversed-phase HPLC, which is helpful to deduce the isomeric structures of the glucosylated PA-glycans [33].

**Fig 7:**
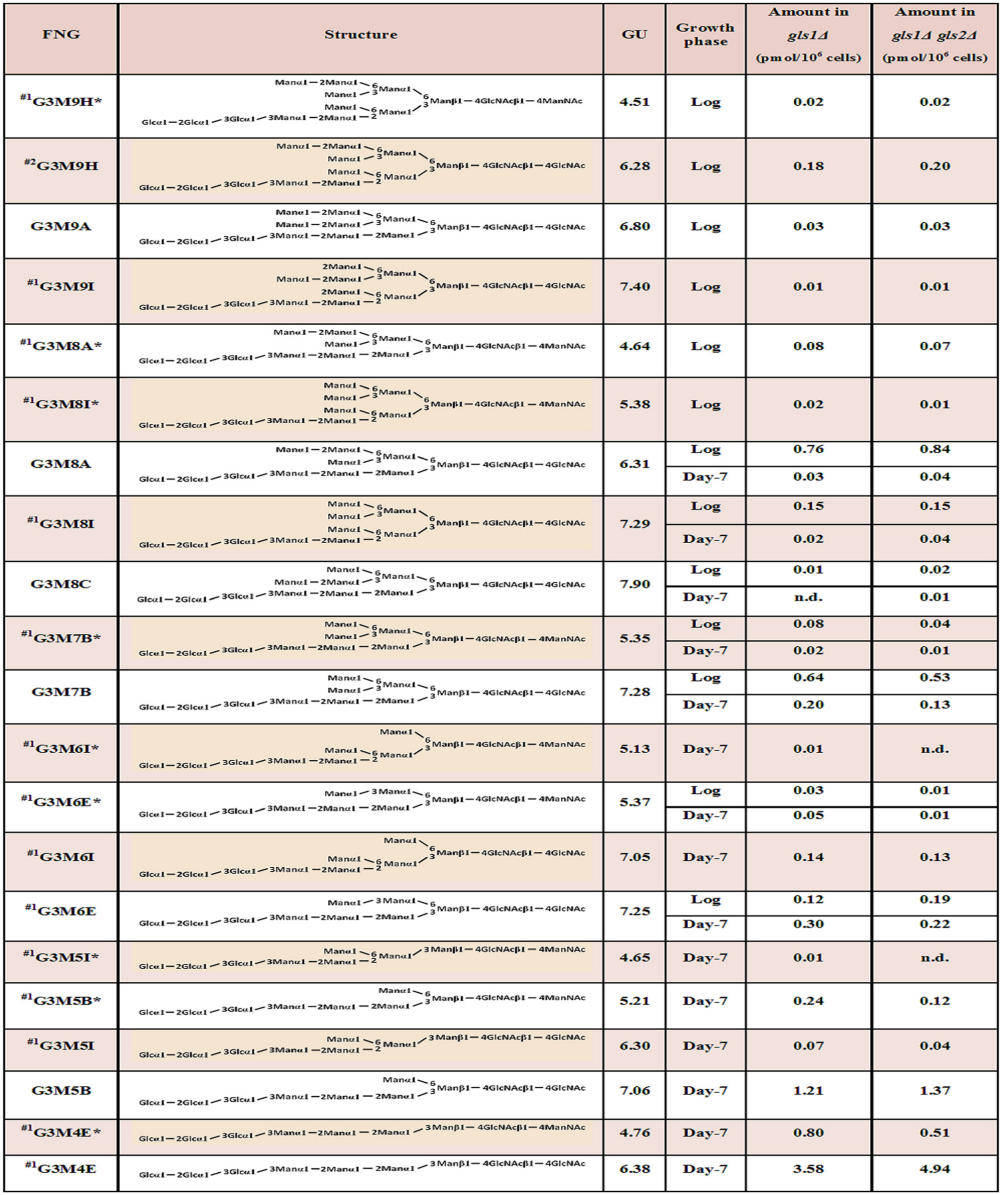
Structures and quantities of PA-labeled FNGs extracted from *gls1*Δ and *gls1*Δ *gls2*Δ cells. The nomenclatures of the FNGs are essentially according to those suggested by Yanagida *et al*. [34]. The Och1-modified FNGs (with the letters 'H' and 'I') were named according to Hirayama *et al*. [12]. n.d.: not determined (<0.01 pmol/10^6^ cells). *: Structure with ManNAc at the reducing terminus. ^#1^: No standard PA-glycans were available; the structures were deduced based on the GU values. ^#2^: Structure was determined based on the GU value of the non-glucosylated glycans reported previously [12].

The FNG isomers were found susceptible to Gls1-only microsome but resistant to Gls2-only microsome, again indicating the presence of the three glucose molecules. We then deduced the structures of the FNG-isomers detected. The structures G3M5B, G3M7B, G3M8A, G3M8C, and G3M9A were confirmed by comparing their elution positions with those of the corresponding authentic PA-sugars in reversed-phase HPLC (S4 Fig). For the structure G3M9H, the respective peak was first completely deglucosylated by treatment with Gls1-only and Gls2-only microsomes and then injected in reversed-phase HPLC. Elution position of this deglucosylated FNG-isomer matched to that of the non-glucosylated M9H-FNG produced in wild type cells as previously reported [12]. The deduced structures of the rest of the isomers could not be unequivocally confirmed due to the lack of authentic standards, as well as resistance of the innermost glucose residue to Gls2-only microsome (S5 Fig; also see ‘Analysis of FNGs in *gls2*Δ cells’ below). Upon treatment with Gls1- and Gls2-only microsomes, the FNG isomers containing the C-arm external mannose (residue 11 in Fig 1) (G3M9H, G3M9A, and G3M8A) showed three GU shift, suggesting complete deglucosylation. On the other hand, only two GU shift was observed for the FNG structures lacking the C-arm α-1,2-linked mannose (all FNG isomers except G3M9H, G3M9A, and G3M8A), suggesting that the innermost glucose was left uncleaved. This result was found to be due to the *in vitro* substrate specificity of Gls2-only microsomes (see below). Nevertheless, considering that all FNGs detected has Gls1-sensitive α-1,2-linked glucose residue (residue 1 in Fig 1), structures of the remaining FNG isomers have been deduced with confidence, especially by comparing the data with those for non-glucosylated FNGs in wild type, *i*.*e*. the elution profile of reversed phase HPLC for Hex_n_HexNAc_2_-PA in *gls1*Δ cells were similar to that of Hex_n-3_HexNAc_2_-PA in wild type cells [12]. The structures and amounts of the FNGs in the *gls1*Δ and *gls1*Δ *gls2*Δ cells thus determined are summarized in Fig 7. FNG isomers with ManNAc residues at the reducing termini were also identified (Figs 5 and 6). The ManNAc residues result from epimerization of the *N*-acetyl group of the reducing-end GlcNAc during reductive amination in the PA-labeling process [12,25]. Isomeric structures of FNGs with the reducing-end ManNAc were predicted based on their GU relative to the GU of equivalent structures with reducing-end GlcNAc. The ‘ManNAc structures’ eluted 1.63–1.93 GU earlier than the corresponding ‘GlcNAc structures’. To confirm the presence of the ManNAc residues at the reducing end of these FNGs, the respective peaks, after isolation by revered-phase HPLC, were digested successively with endo-α-mannosidase and Jack bean α-mannosidase therefore converting these isomers into Man_1_GlcNAc_1_ManNAc_1_ (S6 Fig).

No Man_1_GlcNAc_2_ or any other deglucosylated glycan, however, was detected either in the log or stationary phase of the *gls1*Δ and *gls1*Δ *gls2*Δ cells (Figs 3 and 4, first and second panels). We therefore conclude that an additional α-1,2-glucosidase is not present in these mutants.

### Analysis of FNGs in *gls2*Δ cells

**Fig 8:**
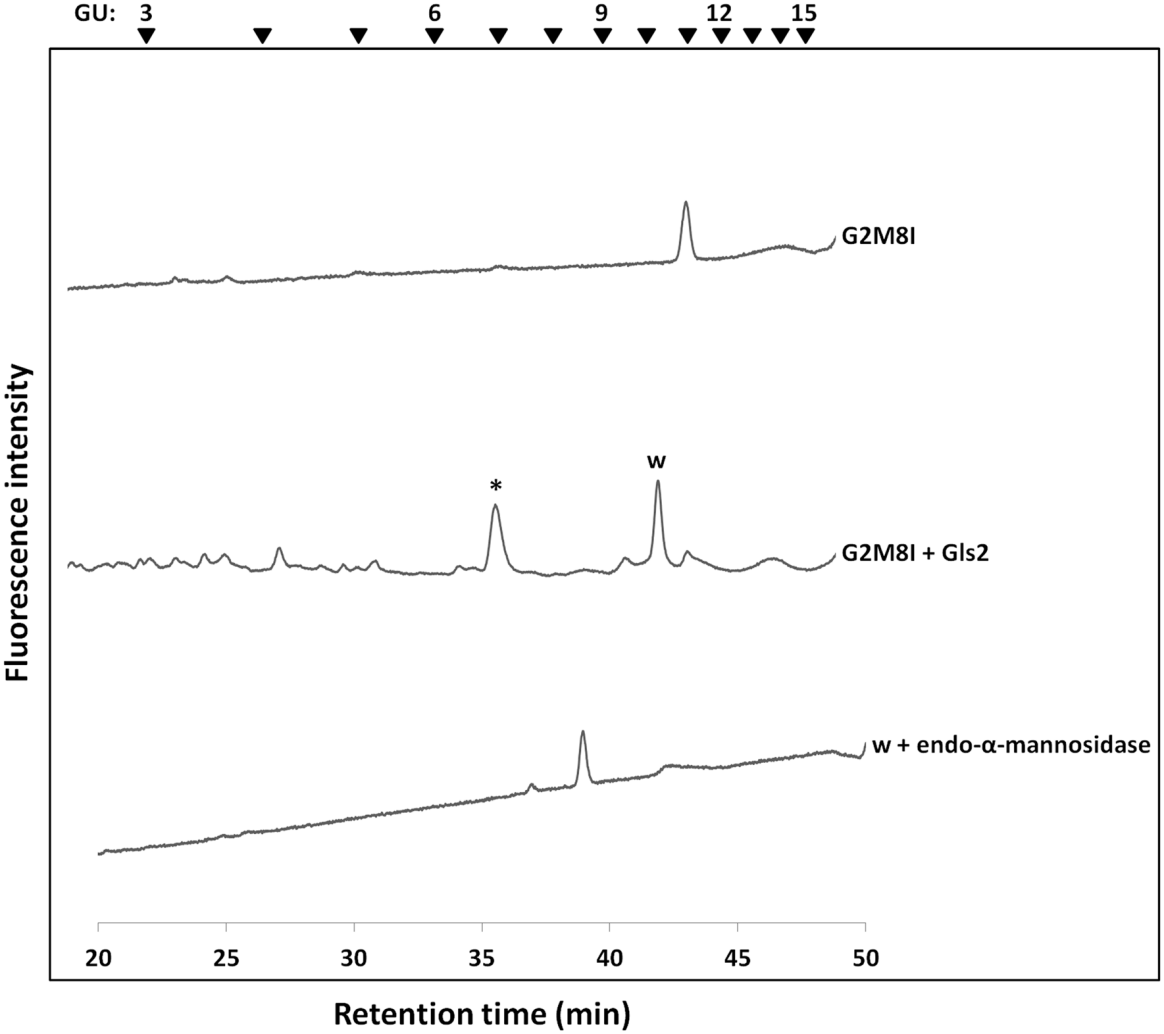
Resistance of the innermost glucose molecule to Gls2-only microsome in G2M8I. The peak corresponding to G2M8I was treated with Gls2-only microsome which resulted in one GU shift in size fractionation HPLC. The resulting peak ‘w’ was collected and treated with endo-α-mannosidase which further shifted the elution position earlier by two GUs, thus indicating the presence of the innermost glucose molecule in ‘w’. The asterisk (*) indicates a contaminating peak appearing in all microsome-treated samples at GU ~7.0 in size fractionation HPLC. The arrowheads indicate the elution position of PA-glucose oligomer for elution standards.

**Fig 9:**
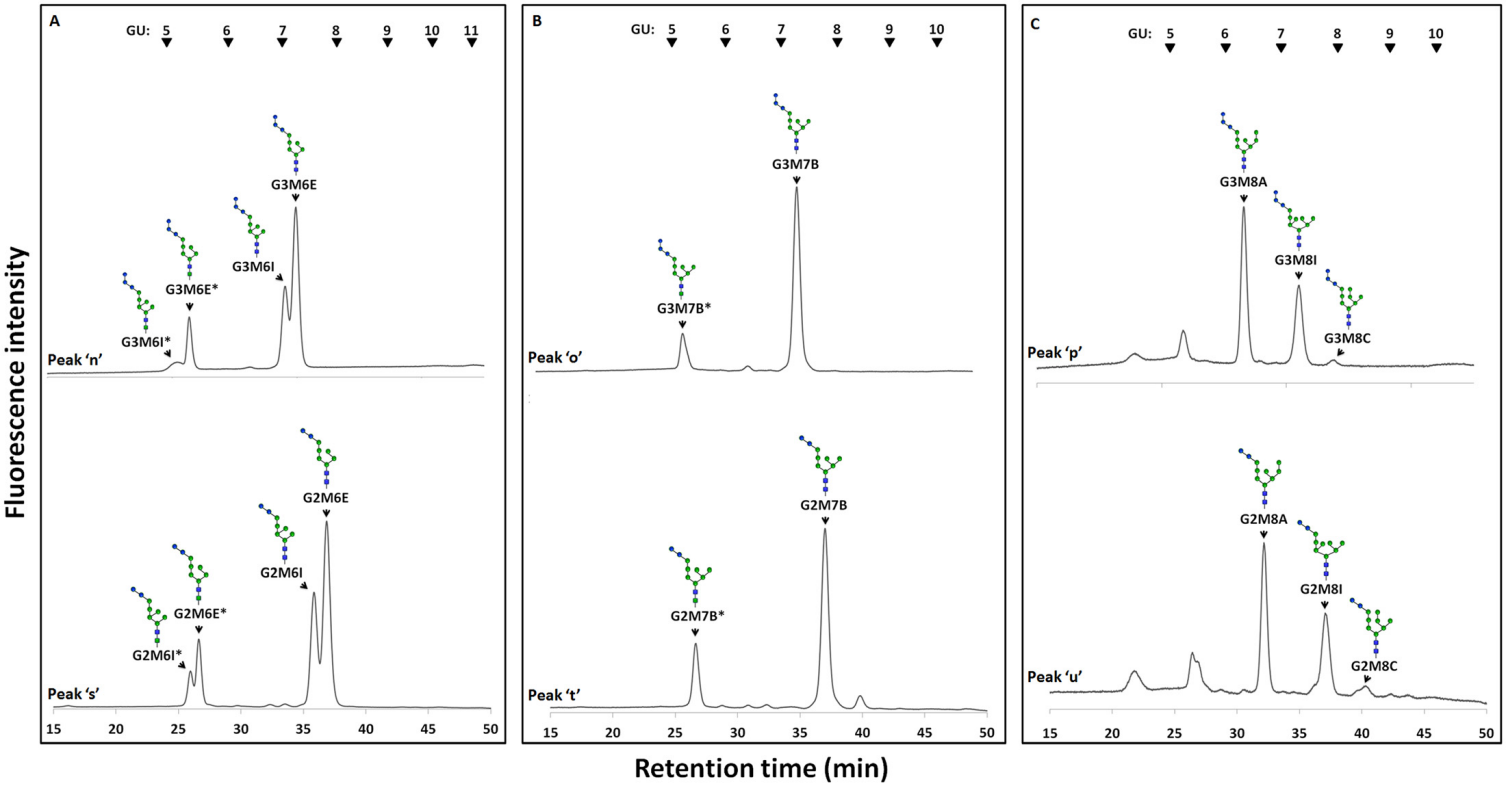
Reversed-phase HPLC profile of tri- and di- glucosylated Man_6_GlcNAc_2_ (A), Man_7_GlcNAc_2_ (B), and Man_8_GlcNAc_2_ (C). The tri- and di- glucosylated FNGs (peaks 'n, o, p' and peaks 's, t, u' respectively) produced in the stationary phase of *gls1*Δ and *gls2*Δ cells respectively, were isolated by size fractionation HPLC and structural isomers of each were further separated in reversed-phase HPLC. Asterisks (*) indicate the structures with ManNAc residues at the reducing end which are produced by GlcNAc-to-ManNAc epimerization of the *N*-acetyl group during PA-labeling reaction. The arrowheads indicate the elution position of PA-glucose oligomer for elution standards.

**Fig 10:**
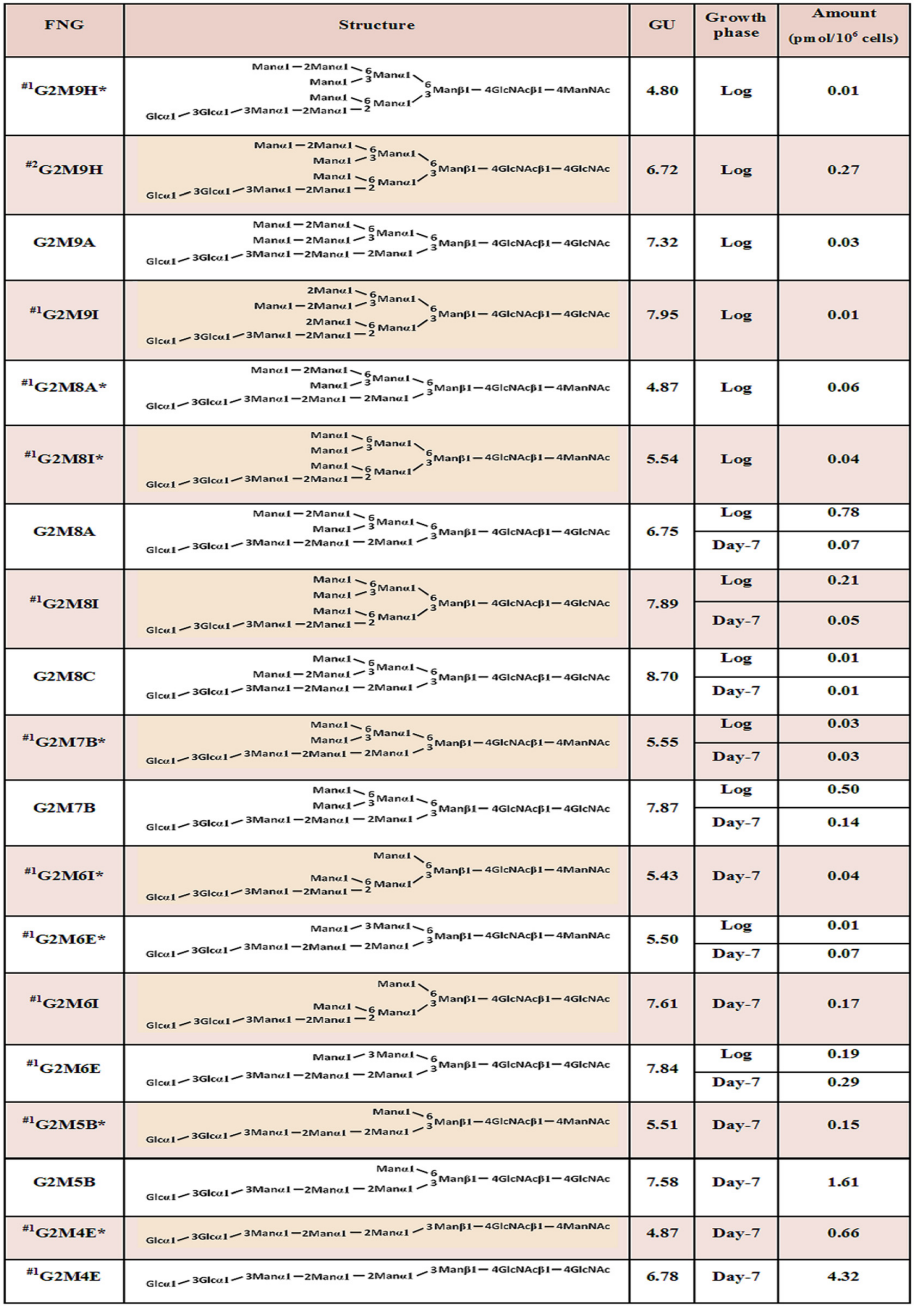
Structures and quantities of PA-labeled FNGs in the log and stationary phases of *gls2*Δ cells. *: Structure with ManNAc at the reducing terminus. ^#^: Structure was confirmed as standard PA-glycans are available.

Since the external α-1,2 linked glucose has been found to be present on the top of all FNGs in *gls1*Δ and *gls1*Δ *gls2*Δ cells, even if an additional α-1,3 glucosidase was present, this enzyme was not able to work on the interior glucose molecules. Therefore, to examine the presence of any novel α-1,3 glucosidase in yeast, next we analyzed FNGs in *gls2*Δ cells. The presence of Gls1 in these cells will result in the removal of the outermost glucose molecule leaving the two interior glucose residues intact, unless any novel α-1,3 glucosidase cleaves those. The peaks ‘h-k’ from the log phase and ‘q-u’ from the stationary phase (Figs 3 and 4, bottom panel) of the *gls2*Δ cells were isolated and each peak was injected in reversed-phase HPLC to separate the FNG isomers. Each isomer was then treated with Gls2-only microsome to examine the number of glucose molecules present. Surprisingly, most of the peaks after microsome treatment showed one GU shift in size fractionation HPLC indicating the removal of only one glucose molecule by the microsome. This suggests that either (1) the FNGs extracted from *gls2*Δ cells were monoglucosylated, from which the innermost glucose molecule was removed by Gls2-only microsome, or (2) the FNGs were diglucosylated and the middle glucose (residue 2 in Fig 1) has been removed by Gls2-only microsome while the innermost glucose (residue 3 in Fig 1) is resistant to the microsome treatment. The second hypothesis, however, is consistent with our finding with the *gls1*Δ and *gls1*Δ *gls2*Δ cells, where the innermost glucose in most of the isomers (those without the C-arm external mannose), were found resistant to Gls2-only microsome. Resistance of the innermost glucose was also evident as the FNGs, already treated with Gls2-only microsome, were still found susceptible to endo-α-mannosidase [17] (Fig 8). It also was found that the reversed-phase HPLC profile of Hex_n_GlcNAc_2_ peaks, obtained from size fractionation HPLC, in *gls1*Δ cells was remarkably similar to that of Hex_n-1_GlcNAc_2_ peaks in *gls2*Δ cells (Fig 9 and S7 Fig). Based on these observations, the isomeric structures of the FNGs in *gls2*Δ cells were determined (Fig 10). On the other hand, FNGs which possessed the terminal mannose residue on C-arm (residue 11 in Fig 1) (G2M9H, G2M9A, and G2M8A) were found to be completely deglucosylated upon Gls2-only microsome treatment, and therefore their structures have been unequivocally determined based on the GU values of nonglucosylated glycans [12].

Again, we were not able to detect Man_1_GlcNAc_2_ or any other deglucosylated glycans in the *gls2*Δ cells as well (Figs 3 and 4, bottom panel). Hence, there is no evidence for the presence of any novel α-glucosidase that acts on *N*-glycans in this yeast.

## Discussion

In this study we examined the issue of whether any novel α-glucosidase is produced in *S*. *cerevisiae* by analyzing the FNG structures produced in α-glucosidase deletion mutants, *gls1*Δ, *gls2*Δ and *gls1*Δ *gls2*Δ. We previously examined FNGs in the wild type cells [12,15] and found that Man_6-9_GlcNAc_2_-FNGs are produced in the log phase whereas all of these high-mannose FNGs are demannosylated in the stationary phase by Ams1 to produce Man_1_GlcNAc_2_, the final enzymatic product of FNG catabolism [15]. In the present study, we found di- or tri-glucosylated FNG structures (Glc_2/3_Man_6-9_GlcNAc_2_) in the log phase of glucosidase deletion mutants whereas in the stationary phase, Glc_2/3_Man_4-8_GlcNAc_2_-FNGs were found, with Glc_2/3_Man_4_GlcNAc_2_ being the final product. However, none of these cells produced any Man_1_GlcNAc_2_ or any other deglucosylated FNGs in either the log or stationary phase cells. These results clearly indicate that *S*. *cerevisiae* produces no additional α-glucosidases that act on the *N*-glycans.

Recently Chantret *et al*. examined FNGs in day-6 cultures of *gls1*Δ and *gls2*Δ cells [16] and, as expected, they observed the production of Glc_3_Man_4-8_GlcNAc_2_ and Glc_2_Man_4-8_GlcNAc_2_-FNGs respectively in these cells. However, they also reported the detection of small amounts of Man_1_GlcNAc_2_ which is unusual to be produced in the absence of the glucosidases, Gls1 and Gls2, unless some type of additional α-glucosidase is present. In any event, our observation is generally consistent with their results as they detected Man_1_GlcNAc_2_ in the glucosidase mutants as a very minute portion of FNGs. It can therefore be feasible to assume that, even if there is a route for the formation of Man_1_GlcNAc_2_ in the stationary phase cells of glucosidase mutants, the process would be extremely inefficient. As they detected the FNGs using metabolic radiolabeling techniques, lack of detection in the current study may just reflect the sensitivity of analytical methods.

What then would be the potential mechanism by which Man_1_GlcNAc_2_ could be formed in the glucosidase mutants, even inefficiently? One obvious possibility is the non-enzymatic degradation of FNGs. For instance, the hydroxyl free radical-mediated cleavage of *N*-glycans has been reported [35]. If a similar radical-mediated cleavage of the FNGs were possible under certain conditions, the formation of Man_1_GlcNAc_2_ in the absence of glucosidases may be possible. Another distant possibility is that this trisaccharide can be produced through the degradation of dolichol-linked oligosaccharides. It was recently reported that, under conditions of glucose-starvation, the biosynthesis of dolichol-linked oligosaccharides is impaired and the phosphorylated FNGs (Man_0-7_GlcNAc_2_-P) can be released from the biosynthetic intermediates of dolichol-linked oligosaccharides via the action of a pyrophosphatase in mammalian cells [26]. The release of phosphorylated FNGs from dolichol-linked oligosaccharides via pyrophosphatase activity has been detected in the microsomes of this yeast [36]. If the non-glucosylated, phosphorylated FNGs were somehow released from the biosynthetic intermediates of dolichol-linked oligosaccharides, Man_1_GlcNAc_2_ might possibly be formed as a catabolic product from the phosphorylated FNGs, and such a possibility should be explored in future studies.

Although, in wild type cells, the predominant FNG found in the stationary phase was Man_1_GlcNAc_2_, in the glucosidase deletion mutants, a significant amount of FNGs higher than the final FNG (Glc_2/3_Man_4_GlcNAc_2_) still remained. This indicates that the demannosylation activity of Ams1 is not as efficient on the glucosylated FNGs as it is for the nonglucosylated FNGs in wild type cells, most probably due to steric hindrance imposed by the glucose molecules.

It is interesting to note that the Gls2-only microsome-mediated deglucosylation of the FNGs showed a strict substrate specificity, and the terminal α-1,2-linked mannose molecule (residue 11 in Fig 1) on C-arm appears to be an absolute requirement for the *in vitro* action of Gls2 on the innermost glucose, under our experimental conditions. This result is somewhat consistent with the observation that mannose trimming on C-arms resulted in reduction of Gls2 activity of rat enzyme [37]. Moreover, it has been shown that this mannose residue is critical for the binding to β subunit of human ER α-glucosidase 2 [38]. However, the Gls2 does not appear to show such strict specificity *in vivo*, as significant amount of non-glucosylated glycans can be observed on glycoproteins even in mutants such as *alg9* [39] or *alg12* in *S*. *cerevisiae* [40], and *alg9/alg12/alg3* in *Schizosaccharomyces pombe* [41]. How such relaxed specificity of Gls2 *in vivo* is achieved, despite its strict specificity *in vitro*, poses an outstanding question that remains to be clarified.

In conclusion, no deglucosylation of FNGs was detected in glucosidase deletion mutant yeast cells and therefore no evidence was obtained to suggest the presence of any new type of α-glucosidase in *S*. *cerevisiae*.

## Supporting Information

S1 Fig Reducing end analysis of Man_1_GlcNAc_1_ManNAc_1_-PA. Man_1_GlcNAc_1_ManNAc_1_-PA is produced by epimerization of the reducing-end GlcNAc of Man_1_GlcNAc_2_ to ManNAc during PA-labeling. Man_1_GlcNAc_2_-PA and Man_1_GlcNAc_1_ManNAc_1_-PA, prepared from wild type yeast, were collected by size fractionation HPLC (A), separated by reversed-phase HPLC (B), and the presence of GlcNAc and ManNAc at the reducing end respectively was confirmed by reducing end analysis (C). Arrowheads indicate the elution positions of authentic PA-ManNAc and PA-GlcNAc.
(TIF)

S2 Fig Ineffectiveness of Gls2- and Gls1- only microsome on PA-G3M9A’ and PA-G2M9A’ respectively. PA-G3M9A’ and PA-G2M9A’ were not deglucosylated upon treatment with Gls2- and Gls1- only microsome respectively. Size-fractionation HPLC profiles are shown. The arrowheads indicate the elution position of PA-glucose oligomer for elution standards; (top panel) profile of PA-G3M9A’. (2^nd^ panel) profile of PA-G3M9A’ digested with Gls2-only microsome. (3^rd^ panel) profile of PA-G2M9A’. (4^th^ panel) profile of PA-G2M9A’ digested with Gls1-only microsome.
(TIF)

S3 Fig Resistance of the peaks ‘a-c’ from glucosidase mutant cells to Gls1- and Gls-2 only microsomes. The three peaks ‘a-c’, observed in the log phase of glucosidase mutant cells, were collected by size fractionation HPLC and each was treated separately by both Gls1- and Gls2- only microsomes. The asterisk (*) indicates a contaminating peak appearing in all microsome treated samples at GU ~7.0. The arrowheads indicate the elution position of PA-glucose oligomer for elution standards.
(TIF)

S4 Fig Confirmation of the predicted FNG structures, G3M5B, G3M7B, G3M8A, G3M8C, and G3M9A by using authentic standards. The FNG isomers predicted to be G3M5B (A), G3M7B (B), G3M8A and G3M8C (C), and G3M9A (D) from the *gls1*Δ *gls2*Δ cells eluted at the same time with the corresponding PA-labeled standard (^std^) glycans in reversed-phase HPLC thus confirming their structures. The arrowheads indicate the elution position of PA-glucose oligomer for elution standards.
(TIF)

S5 Fig Requirement of C-arm external mannose for the activity of Gls2-only microsome on the innermost glucose. Gls1- and Gls2- microsomes removed all the three glucose residues from G3M8A and G3M9H, both having the C-arm external mannose (A), but left the innermost glucose uncleaved in G3M4E and G3M8I which lack that C-arm mannose (B). Size fractionation HPLC profiles are shown. The asterisk (*) indicates a contaminating peak appearing in all microsome treated samples at GU ~7.0. The arrowheads indicate the elution position of PA-glucose oligomer for elution standards.
(TIF)

S6 Fig Confirmation ManNAc residue at the reducing end. The FNG isomers, G2M6E* and G2M7B*, predicted to have ManNAc molecules at the reducing end, were collected by reversed-phase HPLC and digested with endo-α-mannosidase followed by Jack bean α-mannosidase to remove all the α-linked hexoses. Re-injection in reversed-phase HPLC produced a peak at the same elution position as Man_1_GlcNAc_1_ManNAc_1_ (cf. see S1 Fig). The arrowheads indicate the elution position of PA-glucose oligomer for elution standards.
(TIF)

S7 Fig Reversed-phase HPLC profile of tri- and di- glucosylated Man_6_GlcNAc_2_ (A), Man_7_GlcNAc_2_ (B), and Man_8_GlcNAc_2_ (C) in the log phase. The tri- and di- glucosylated FNGs (peaks 'd, e, f' and peaks 'h, i, j' respectively) produced in the log phase of *gls1*Δ and *gls2*Δ cells respectively, were isolated by size fractionation HPLC and re-injected in reversed-phase HPLC. Asterisks (*) indicate the structures with ManNAc residues at the reducing end. The arrowheads indicate the elution position of PA-glucose oligomer for elution standards.
(TIF)
