# Supplementary figures and images for: Structural Analysis of Free N-Glycans in α-Glucosidase Mutants of Saccharomyces cerevisiae: Lack of the Evidence for the Occurrence of Catabolic α-Glucosidase Acting on the N-Glycans
Source: PLoS One. 2016 Mar 24;11(3):e0151891. doi: 10.1371/journal.pone.0151891 (PMC4807098; doi:10.1371/journal.pone.0151891)

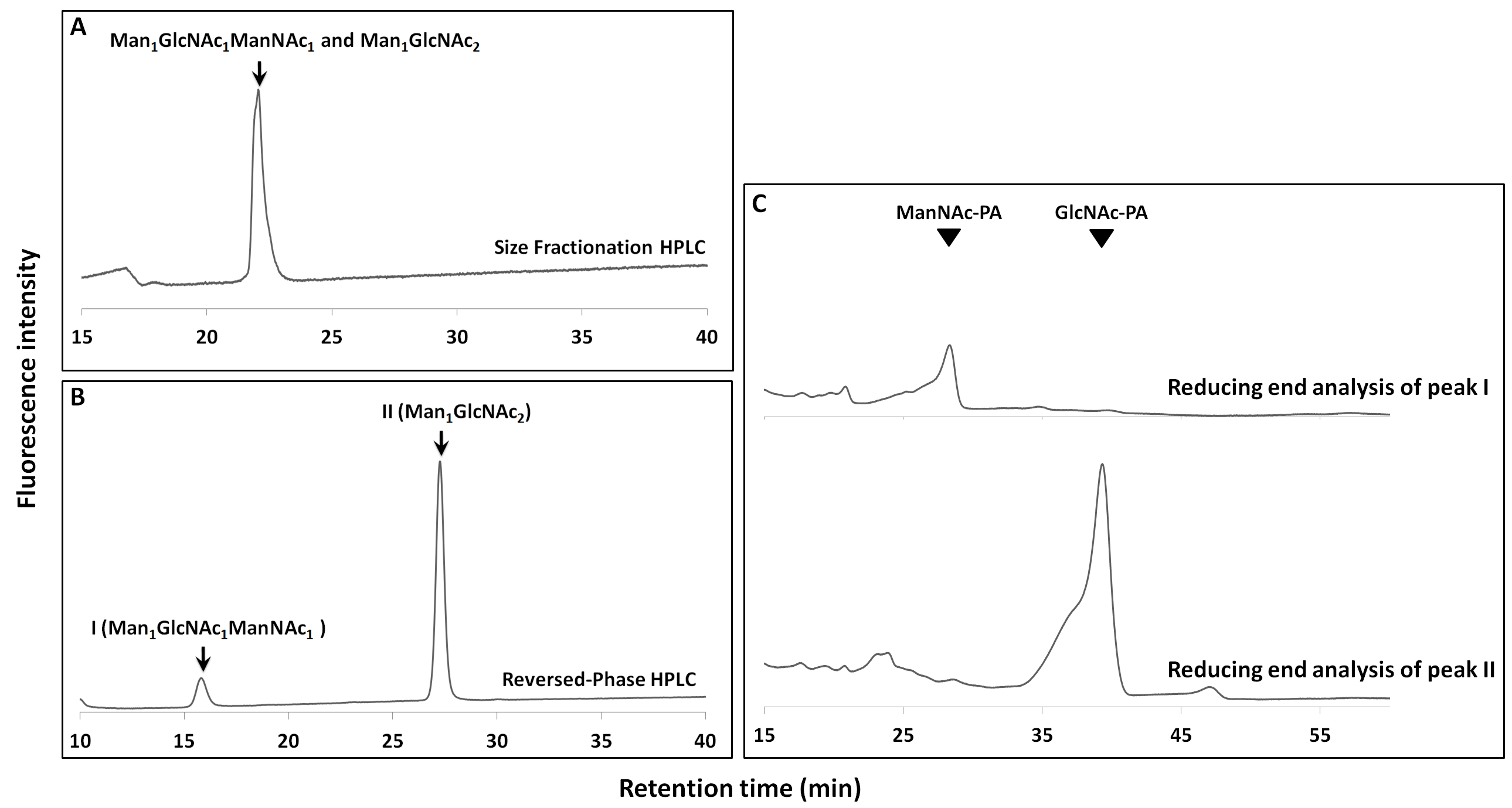

Supplement: S1 Fig — Man1GlcNAc1ManNAc1-PA is produced by epimerization of the reducing-end GlcNAc of Man1GlcNAc2 to ManNAc during PA-labeling. Man1GlcNAc2-PA and Man1GlcNAc1ManNAc1-PA, prepared from wild type yeast, were collected by size fractionation HPLC (A), separated by reversed-phase HPLC (B), and the presence of GlcNAc and ManNAc at the reducing end respectively was confirmed by reducing end analysis (C). Arrowheads indicate the elution positions of authentic PA-ManNAc and PA-GlcNAc. (TIF) [file pone.0151891.s001.tif]

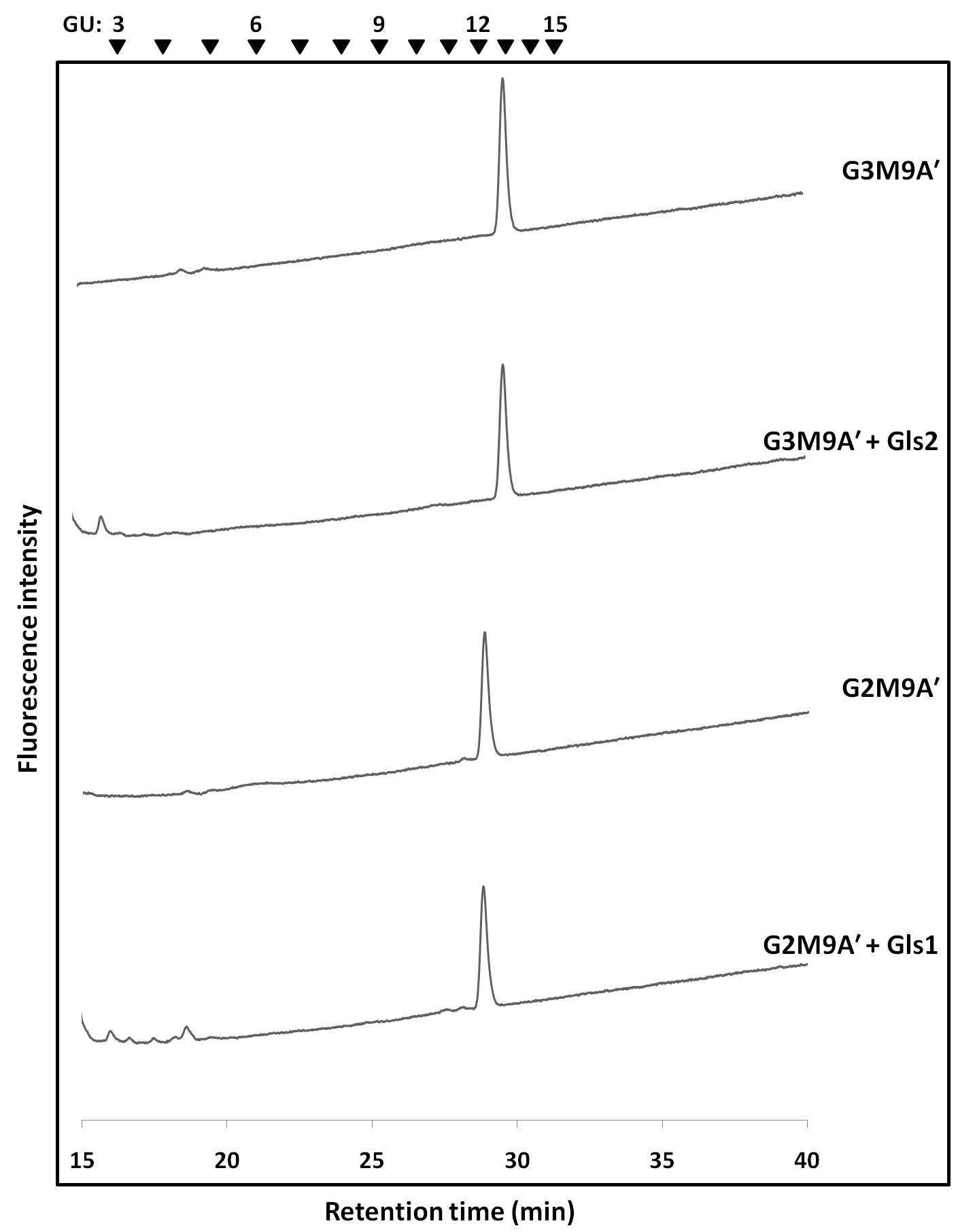

Supplement: S2 Fig — PA-G3M9A’ and PA-G2M9A’ were not deglucosylated upon treatment with Gls2- and Gls1- only microsome respectively. Size-fractionation HPLC profiles are shown. The arrowheads indicate the elution position of PA-glucose oligomer for elution standards; (top panel) profile of PA-G3M9A’. (2nd panel) profile of PA-G3M9A’ digested with Gls2-only microsome. (3rd panel) profile of PA-G2M9A’. (4th panel) profile of PA-G2M9A’ digested with Gls1-only microsome. (TIF) [file pone.0151891.s002.tif]

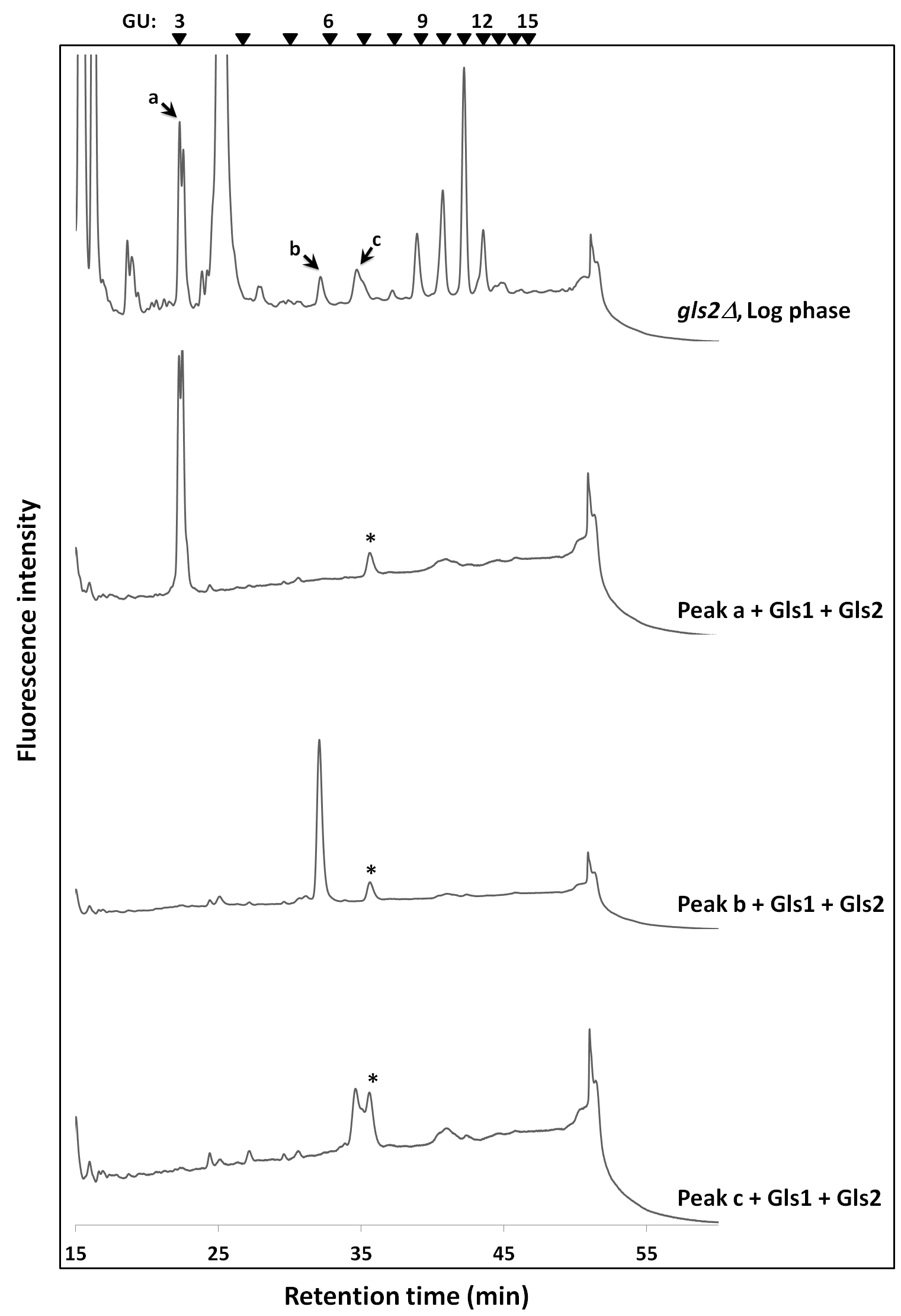

Supplement: S3 Fig — The three peaks ‘a-c’, observed in the log phase of glucosidase mutant cells, were collected by size fractionation HPLC and each was treated separately by both Gls1- and Gls2- only microsomes. The asterisk (*) indicates a contaminating peak appearing in all microsome treated samples at GU ~7.0. The arrowheads indicate the elution position of PA-glucose oligomer for elution standards. (TIF) [file pone.0151891.s003.tif]

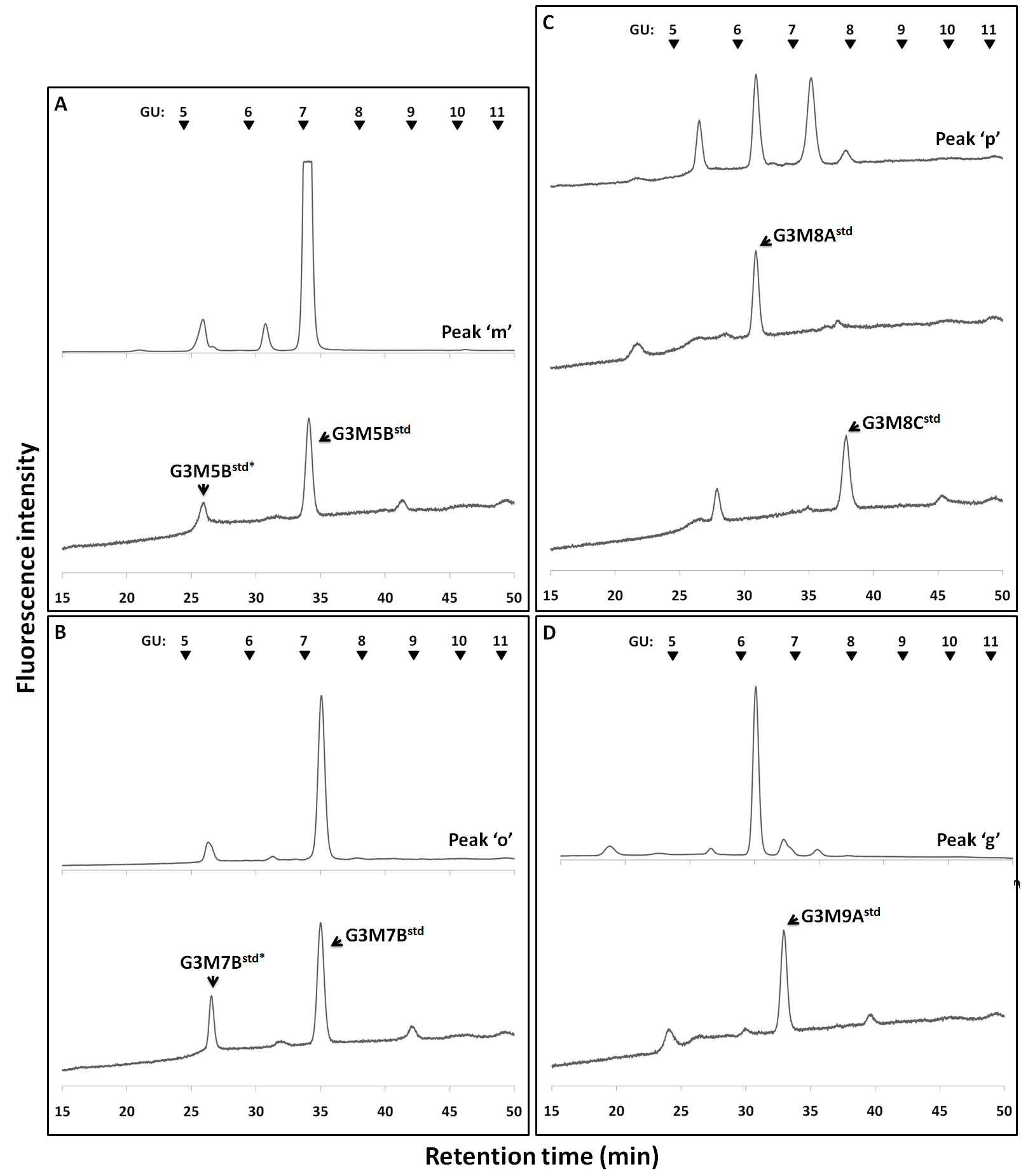

Supplement: S4 Fig — The FNG isomers predicted to be G3M5B (A), G3M7B (B), G3M8A and G3M8C (C), and G3M9A (D) from the gls1Δ gls2Δ cells eluted at the same time with the corresponding PA-labeled standard (std) glycans in reversed-phase HPLC thus confirming their structures. The arrowheads indicate the elution position of PA-glucose oligomer for elution standards. (TIF) [file pone.0151891.s004.tif]

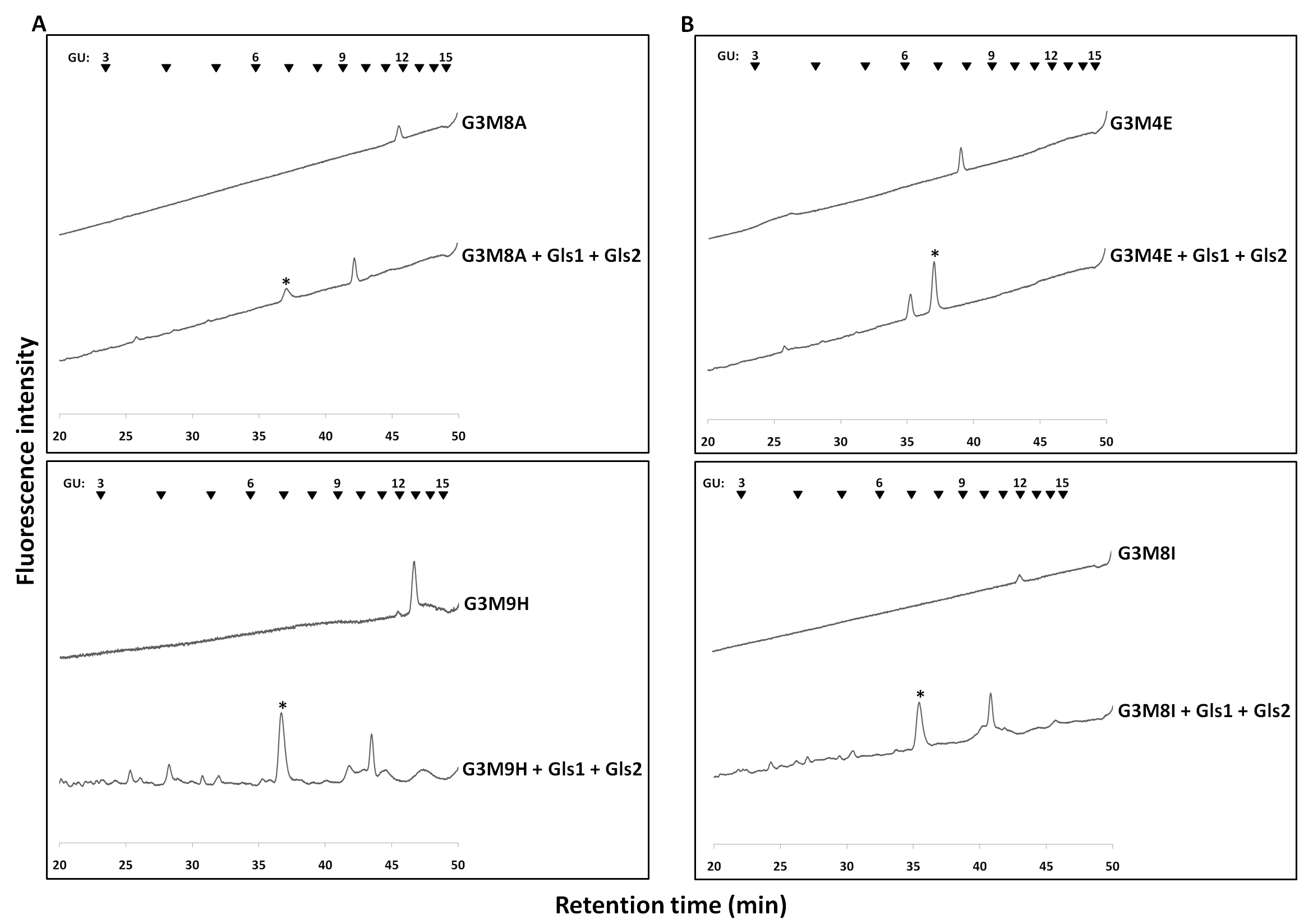

Supplement: S5 Fig — Gls1- and Gls2- microsomes removed all the three glucose residues from G3M8A and G3M9H, both having the C-arm external mannose (A), but left the innermost glucose uncleaved in G3M4E and G3M8I which lack that C-arm mannose (B). Size fractionation HPLC profiles are shown. The asterisk (*) indicates a contaminating peak appearing in all microsome treated samples at GU ~7.0. The arrowheads indicate the elution position of PA-glucose oligomer for elution standards. (TIF) [file pone.0151891.s005.tif]

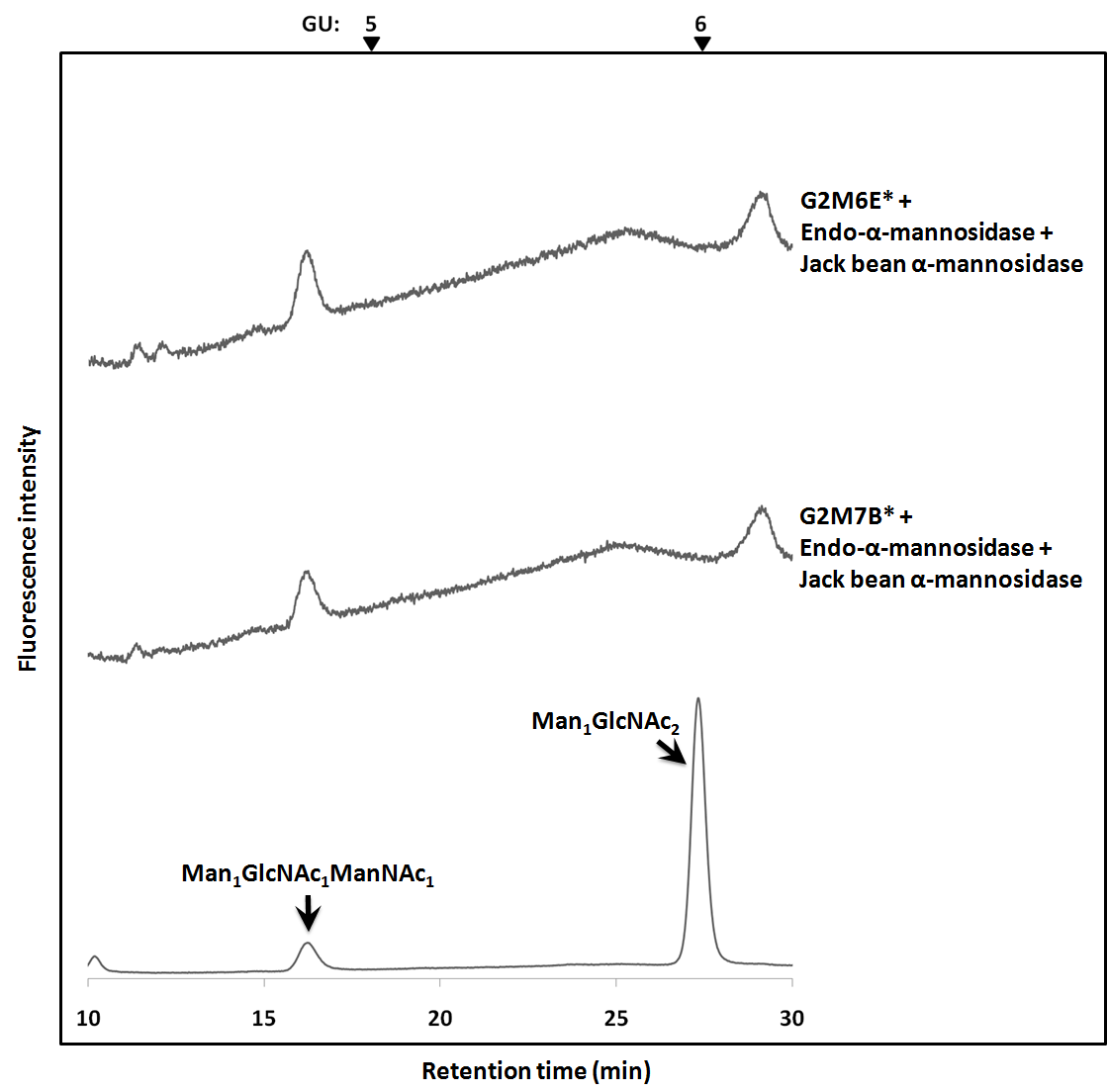

Supplement: S6 Fig — The FNG isomers, G2M6E* and G2M7B*, predicted to have ManNAc molecules at the reducing end, were collected by reversed-phase HPLC and digested with endo-α-mannosidase followed by Jack bean α-mannosidase to remove all the α-linked hexoses. Re-injection in reversed-phase HPLC produced a peak at the same elution position as Man1GlcNAc1ManNAc1 (cf. see S1 Fig). The arrowheads indicate the elution position of PA-glucose oligomer for elution standards. (TIF) [file pone.0151891.s006.tif]

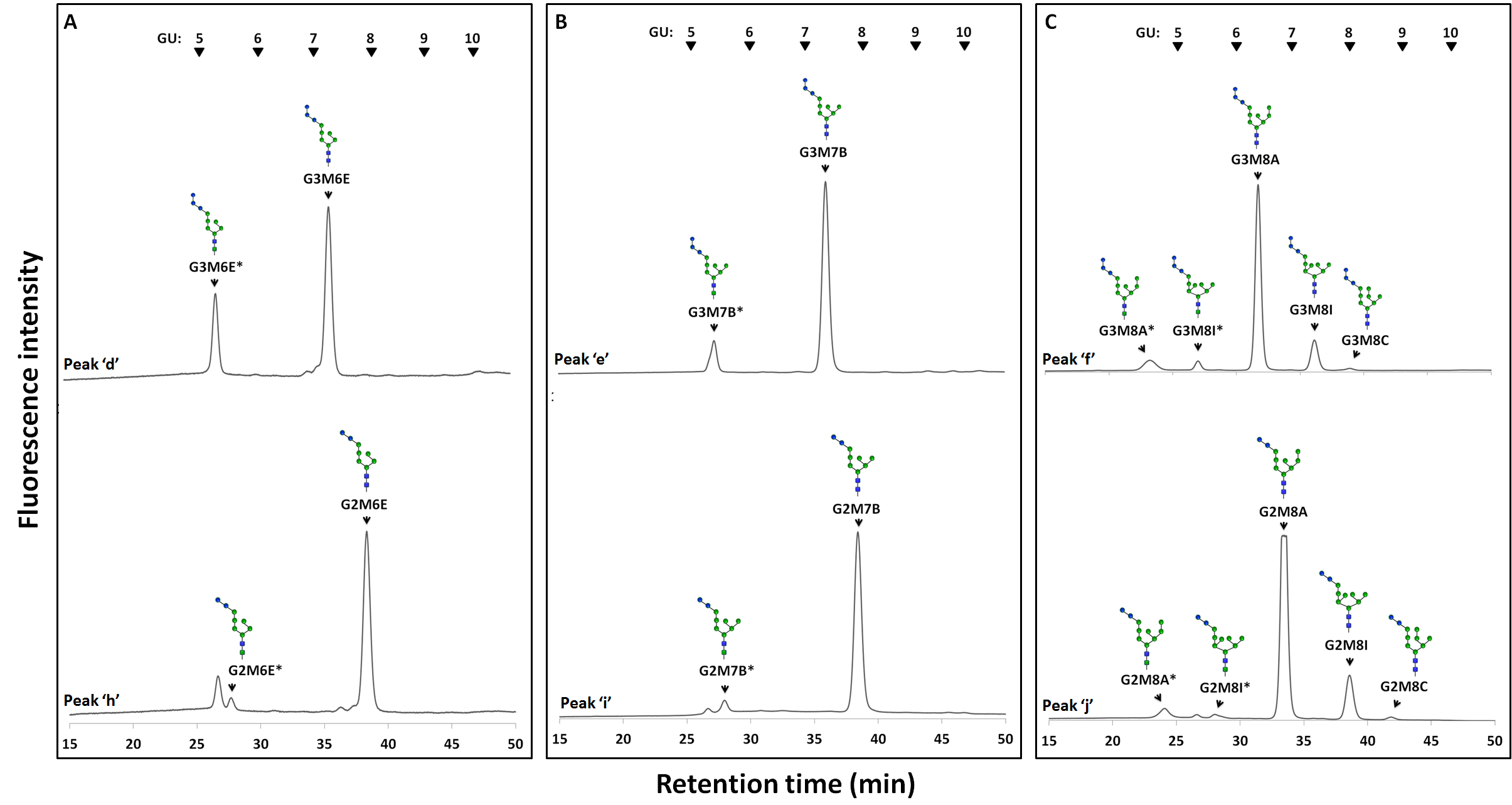

Supplement: S7 Fig — The tri- and di- glucosylated FNGs (peaks 'd, e, f' and peaks 'h, i, j' respectively) produced in the log phase of gls1Δ and gls2Δ cells respectively, were isolated by size fractionation HPLC and re-injected in reversed-phase HPLC. Asterisks (*) indicate the structures with ManNAc residues at the reducing end. The arrowheads indicate the elution position of PA-glucose oligomer for elution standards. (TIF) [file pone.0151891.s007.tif]
